# Reporting of molecular test results from cell-free DNA analyses: expert consensus recommendations from the 2023 European Liquid Biopsy Society ctDNA Workshop

**DOI:** 10.1016/j.ebiom.2025.105636

**Published:** 2025-03-22

**Authors:** Vincent D. de Jager, Patrizio Giacomini, Jennifer A. Fairley, Rodrigo A. Toledo, Simon J. Patton, Simon A. Joosse, Claudia Koch, Zandra C. Deans, ELBS ctDNA Workshop Group, Sofia Agelaki, Claus Lindbjerg Andersen, Daniel Andersson, Beatriz Bellosillo, Inger Riise Bergheim, Daan van den Broek, Zandra C. Deans, Els Dequeker, Jennifer A. Fairley, Beatriz García Peláez, Patrizio Giacomini, Alastair Greystoke, Ariane Hallermayr, Ellen Heitzer, T. Jeroen N. Hiltermann, Michael Hubank, Stefano Indraccolo, Vincent D. de Jager, Simon A. Joosse, Laura Keller, Matthew Krebs, Marjolijn Ligtenberg, Leandro Lo Cascio, Miguel A. Molina-Vila, Krystyna Nahlik, Michael Neumaier, Björn Nowack, Anca Oniscu, Stephan Ossowski, Andre Oszwald, Niels Pallisgaard, Klaus Pantel, Simon J. Patton, Mads Heilskov Rasmussen, Etienne Rouleau, Amit Roshan, Mitja Rot, Helene Schlecht, Ed Schuuring, Ulrich Schüller, Laxmi Silwal-Pandit, Holger Sültmann, Philippe Taniere, Rodrigo Toledo, Nora Wuerdemann, Klaus Pantel, Ellen Heitzer, Ed Schuuring

**Affiliations:** a Department of Pathology and Medical Biology, University Medical Center Groningen, University of Groningen, Groningen, the Netherlands; b UOSD Medicina di Precisione in Senologia, Fondazione Policlinico Universitario Agostino Gemelli IRCCS, Rome, Italy; c Member of the European Liquid Biopsy Society (ELBS) ctDNA Working Group, Hamburg, Germany; d GenQA, Department of Laboratory Medicine, NHS Lothian, Nine, Edinburgh Bioquarter, 9 Little France Road, Edinburgh, EH16 4SA, United Kingdom; e Vall d'Hebron Institute of Oncology (VHIO), Vall d'Hebron Barcelona Hospital Campus, Barcelona, Spain; f EMQN CIC, Unit 4, Enterprise House, Manchester Science Park, Pencroft Way, Manchester, M15 6SE, United Kingdom; g Department of Tumor Biology, University Medical Center Hamburg-Eppendorf, Martinistr. 52, 20246, Hamburg, Germany; h European Liquid Biopsy Society (ELBS), University Medical Center Hamburg-Eppendorf, Martinistraße 52, 20246, Hamburg, Germany; i Institute of Human Genetics, Diagnostic & Research Center for Molecular BioMedicine, Medical University of Graz, Graz, Austria; j Mildred Scheel Cancer Career Center HaTriCS4, University Medical Center Hamburg-Eppendorf, Martinistr. 52, 20246, Hamburg, Germany; k Christian Doppler Laboratory for Liquid Biopsies for Early Detection of Cancer, Medical University of Graz, Graz, Austria

**Keywords:** ctDNA test reporting, Liquid biopsy, Expert consensus recommendations

## Abstract

The implementation of circulating tumor DNA (ctDNA) in the diagnostic routine may enable non-invasive predictive biomarker testing and treatment optimization in patients who lack a suitable tumor specimen, have failed previous molecular analysis or are clinically ineligible for (re-)biopsy procedures. As the interpretation and reporting are more complex for ctDNA than conventional tissue-based NGS, there is a need for specific guidelines. These will offer support for the reporting of ctDNA test results and will facilitate optimal communication of liquid biopsy findings between diagnostic laboratories and the medical oncology team. Aiming to generate guidelines based on real-world experiences and broad perspectives, we organized a European Liquid Biopsy Society (ELBS) ctDNA workshop, in which forty-four experts and key stakeholders from different molecular diagnostics laboratories, oncology and pathology departments, as well as an IVDR specialist, convened to address significant challenges associated with the reporting of liquid biopsy test results. This report delineates the resulting consensus recommendations for ctDNA test reporting with underlying rationale and background information.

**Research in context**

**Evidence before this study**

While guidelines for the reporting of tissue-based next-generation sequencing (NGS) results are already well-established, recommendations for reporting NGS-based molecualr profiling results from circulating tumor DNA (ctDNA) are less advanced. Nevertheless, guidelines primarily aimed at ensuring accuracy, clinical relevance, and clear communication between laboratories and healthcare providers are critical because ctDNA analysis is more complex than traditional tissue-based NGS due to factors like tumor heterogeneity, lower DNA yield, and potential contamination from non-tumor DNA (e.g., hematopoietic variants). Although previous efforts have mostly led to literature-based recommendations on specific elements of reports, practical guidance for reporting of cfDNA-based NGS results remains scarce.

**Added value of this study**

This study—led by the ctDNA technology work group of the European Liquid Biopsy Society (ELBS)—provides the first expert consensus recommendations for reporting cfDNA-based NGS results, drawn from the expertise of a multidisciplinary group of professionals. The development and implementation of standardized guidelines for ctDNA reporting is crucial for enhancing clinical utility and ensuring clear communication between diagnostic laboratories and oncology teams. These consensus recommendations address the complex challenges of ctDNA test interpretation, offering a framework to improve the accuracy and consistency of liquid biopsy reporting.

**Implications of all the available evidence**

This initiative will enable broader adoption of ctDNA in routine diagnostics, providing non-invasive, real-time insights for personalized treatment strategies, especially for patients without access to traditional tissue biopsies or those who are ineligible for repeat biopsies. The recommendations can be harnessed directly by clinical scientists (molecular) pathologists and geneticists for reporting of cfDNA-based test results and support test report interpretation by the treating oncologist.

## Introduction

Precision cancer medicine entails tailored-treatment selection based on specific tumor characteristics and has contributed to the improvement of treatment outcomes of patients with cancer, in particular in non-small cell lung cancer, breast cancer, and colorectal cancer.^1^^,^^2^ With the rapid expansion of clinically relevant biomarkers in various cancer types, genomic profiling is becoming a necessity to guide treatment and the clinical decision-making process in an increasing number of malignancies.^3^, ^4^, ^5^ Although predictive biomarker testing conventionally involves tissue-based testing, in recent years, liquid-based testing, mostly the analysis of circulating tumor DNA (ctDNA), has been translated into the clinic. Initially, single biomarker tests to interrogate single genes or few hotspots in plasma-derived circulating cell-free (ccfDNA) were approved as companion diagnostic for molecular targeted drugs. For example, the PCR-based cobas™ *EGFR* test has been used to detect common *EGFR* mutations and the *EGFR* T790M resistance mutation in plasma-derived ccfDNA of patients with non-small cell lung cancer.^6^ However, with the ever-growing number of actionable gene alterations and pan-cancer biomarkers,^7^^,^^8^ both tissue and ctDNA testing are moving towards comprehensive genomic profiling (CGP), typically carried out with next-generation sequencing (NGS). Unlike traditional single-gene tests, CGP provides a broad picture of the genetic alterations and enables the detection of all main classes of genomic alterations.^9^ Although most CGP tests for ctDNA provide informative results, interpretation is complex and often poses challenges as to how the results of liquid biopsy-based CGP should be reported. ctDNA guidelines are sparse compared to existing recommendations for the interpretation and reporting of tissue-based NGS.^10^, ^11^, ^12^, ^13^, ^14^, ^15^ In 2022, the European Society for Medical Oncology (ESMO) Precision Medicine Working Group convened a group of experts to provide recommendations on various aspect of ctDNA testing based on published literature.^15^ Previously published recommendations offer a valuable framework for reporting of ctDNA tests (see summary in Table 1)^10^, ^11^, ^12^, ^13^, ^14^, ^15^^,^^17^; however, these are mostly based on literature reviews rather than real-world experience and clinical practice and lack guidance and concrete recommendations on appropriate reporting of molecular findings from ccfDNA NGS analysis.

**Table 1:** Essential elements of report of liquid biopsy as previously described in literature.

Element/recommendation	References
Reference to ISO 15189 requirements^16^	^10^^,^^17^
**Pre-analytical variables**	
Unique patient identifier	11, 12, 13^,^^15^
Diagnosis and disease stage	^17^
Patient history	^17^
Requesting person/laboratory	^11^^,^^12^^,^^17^
Sample storage method	^12^
Date of sample collection	^11^^,^^12^^,^^17^
**Test methods**	
Material used for extraction and DNA quantity used for test	11, 12, 13, 14
Test type	^12^^,^^17^
Genes covered	^12^^,^^14^^,^^15^^,^^17^
Investigated variants (if applicable)	^14^^,^^15^^,^^17^
Sensitivity/specificity/limit of detection/certainty of findings of assay	10, 11, 12^,^^14^^,^^15^^,^^17^
**Test results and interpretation**	
Reporting of (likely) pathogenic variants	^11^
Reporting of VUS	^11^^,^^17^
(Likely) benign should not be reported	^11^
Allelic frequency/variant	^10^^,^^11^^,^^15^^,^^17^
Coverage (+- other quality control metrics)	^10^^,^^11^^,^^17^
Variant type (e.g., amplification, mutation)	^10^^,^^15^^,^^17^
Clinical interpretation, clinical significance, actionability of mutation(s)	10, 11, 12^,^^17^
Avoid the use of term ‘wildtype’ for negative liquid biopsy results due to lower sensitivity (as compared to tissue-based testing)	^11^^,^^12^^,^^14^^,^^15^
Potential germline variants	^15^^,^^17^
Potential CH-related variants	^15^

VUS, variant of unknown (clinical) significance; CH, clonal hematopoiesis.

The ctDNA technology working group of the European Liquid Biopsy Society (ELBS) sought to bring together key stakeholders in the liquid biopsy space with hands-on routine expertise, to collect expert opinions on how to best report molecular ctDNA test results to the treating physician. To this end, in October 2023, we organized an interdisciplinary two-day workshop that was attended by forty-four leading experts in molecular biomarker testing from across Europe. During the workshop, experts engaged in discussions addressing key challenges that are encountered when using ctDNA testing in daily clinical practice, and devoted collaborative efforts to establish consensus reporting recommendations for molecular profiling of ctDNA in advanced-stage cancer to detect actionable variants. Based on questionnaires and use cases, reporting recommendations were formulated for predictive biomarker testing using NGS ctDNA testing.

In this manuscript, we provide expert opinion reporting recommendations from real-world experience along with the underlying rationales. Our reporting recommendations are meant to support diagnostic laboratories (molecular) pathologists, and treating physicians in the implementation of ctDNA testing and may help to harmonize result interpretation among different healthcare professionals.

## Methodology

During this in-person ELBS ctDNA workshop, forty-four attending experts including clinical genomic specialists, molecular pathologists, clinical oncologists, clinical chemists, human geneticists, experts in external quality assessment (EQA), reference materials providers and one In Vitro Diagnostics Regulation (IVDR) specialist were organized into groups of five to eight people, promoting open and collaborative discussions. Each group was tasked with discussing the interpretation and reporting of molecular test results, based on a set of clinical use cases. Following group discussions, cases were deliberated in plenary sessions involving all workshop attendees. The organizers (VdJ, PG, JF, RT, SP, SAJ, CK, ZD, EH, ES) collected inputs and opinions shared during these discussions, and summarized them in an online questionnaire including ten topics, each of which is specific for a distinct step of the diagnostic workflow: (1) request for ctDNA testing, (2) availability of historical information prior to ctDNA testing, (3) pre-analytical variables and timeframe, (4) assay specifications and performance assessment, (5) quality metrics, (6) reporting of variants, (7) distinguishing clonal hematopoiesis (CH)-related variants from tumor-derived variants, (8) somatic copy number alterations and fusions, (9) reporting of negative results, and (10) unexpected findings. In order to empirically evaluate the extent of consensus, workshop attendees were invited to provide their level of agreement to each statement by choosing one of four options: ‘agree, essential’, ‘agree, useful’, ‘disagree’ or ‘no opinion’. Additionally, for each statement, respondents had the possibility to provide feedback or remarks. The complete questionnaire is available in Supplementary File S1.

Predetermined cut-offs for the assessment of the level of agreement among the attending experts were used. An agreement of 80% or above was considered as expert opinion consensus, 71–79% as strong expert opinion agreement, 61–70% as weak expert opinion agreement, and below 60% as no agreement reached.^18^ Based on the outcome of this questionnaire, twenty-five ELBS recommendations for ctDNA molecular profiling were formulated (Table 2) and finally reviewed by all experts of the ELBS ctDNA Working Group.

**Table 2:** ELBS expert opinion recommendations on ctDNA reporting.

ELBS expert opinion recommendations on ctDNA reporting		
Intention of the test and patient information	1	Prior to testing, patients should be informed about the potential consequences of unexpected or incidental findings.
	2	Request forms should include an opt-out box in case patients do NOT wish to be informed about unexpected and/or incidental findings.
	3	The purpose for testing should be clearly stated on the request form.
	4	If available, information about pathological diagnosis, disease stage, burden of disease, disease status, previous and current oncological treatment, previously diagnosed malignancies, confirmed tumor predisposition, mutations from previous tissue profiling or liquid profiling, and previously identified CH-related variants, should be provided with the request form.
	5	Relevant available clinical information should be included in the report.
Technical aspects listed on the test report	6	Test QC: Technical assay specifications should be stated in the methods section of the report and include scope and type of the test, LOD, LOB, LOQ, analytical sensitivity, analytical specificity, cfDNA isolation method, percentage of target region covered with the minimum required depth. If any of the listed specifications are not met, this should be clearly stated.
	7	Sample QC: Sample-specific parameters do not need to be included, BUT any deviation affecting sensitivity should be clearly stated, including macroscopic abnormalities (e.g. hemolysis), lower DNA input, contamination with high molecular weight DNA.
	8	Run QC: Run-specific parameters (e.g. base calling quality scores, average sequencing depth, etc.) do not need to be included, BUT if quality metric requirements of the assay are not met, this should be clearly stated.
	9	Variant QC: Detected variants with VAF below or equal to the LOB should NOT be listed.
	10	Variants with VAF between LOB and LOD should be labeled as ‘equivocal’ and accompanied by a disclaimer stating the uncertainty of their presence.
	11	To confirm the presence of equivocal variants, repeated or orthogonal testing should be performed. If the variant remains uncertain, corresponding tissue testing and/or liquid re-biopsy should be recommended.
Tumor-derived & non-tumor-derived variants	12	For each variant, the number of supporting reads, sequencing depth, VAF, number of mutated molecules as well as a confidence level should be reported.
	13	Pathogenic and likely pathogenic variants should be listed in the main report, whereas VUS can be listed either in the main report or as an Appendix.
	14	Variants in cancer susceptibility genes with VAF indicating germline origin should be highlighted as such.
	15	If a putative germline variant is reported, genetic counselling and/or germline testing should be recommended.
	16	Without PBMC-testing to correct for CH-related variants, variants suspected to originate from non-tumor sources should be flagged as a ‘potential CH-related variant’.
SCNA & fusions	17	The report should clearly state that the LOD for SCNA and fusions may be lower compared to SNVs/indels and that their detection requires a higher tumor fraction.
	18	For SCNA, the estimated copy number or log2 ratio, confidence level, potentially co-amplified genes and estimated size of the amplified/deleted segment should be reported.
Negative results	19	If tumor fraction estimation is not included in the tests, negative test results should be reported as ‘not detected’. The use of terms as ‘wildtype’, ‘negative’ or ‘absence of mutation(s)’ should be avoided.
	20	If specific mutations were requested, test results should be reported as ‘requested mutation is not detected’.
	21	Each report should include a disclaimer that the presence of mutations below the LOD cannot be excluded.
Unexpected findings	22	Unexpected findings should be accompanied by a disclaimer, including an explanation why the findings were unexpected.
	22	Unexpected findings should standardly be referred to a Molecular Tumor Board for discussion.
Actionability	24	Clinically actionable results and evidence-based associations with response to specific drugs should be disclosed, but treatment recommendations should not be given.
	25	The actual clinical annotation for matching a treatment to a specific variant for each individual patients should only be done by the treating physician or a Molecular Tumor Board (MTB).

CH, clonal hematopoiesis; ELBS, European Liquid Biopsy Society; cfDNA, cell-free DNA; LOB, limit of blank; LOD, limit of detection; LOQ, limit of quantification; Indel, small insertions and deletions; PBMC, peripheral blood mononuclear cell; QC, quality control; SCNA, somatic copy number alterations; SNV, single nucleotide variant; VAF, variant allele fraction, VUS, variant of uncertain significance.

Since the focus of the workshop was on diagnostic reporting rather than on technical aspects, modalities for performance assessment of ctDNA test were not discussed in detail. Yet, there was consensus among all experts that as with tissue-based NGS, diagnostic laboratories performing ctDNA-based NGS should adhere to the ISO 15189 standards and existing guidelines, which provide an overview of the minimal requirements for pathology reports, including reports of molecular tests.^16^

### Ethics statement

Formal institutional review board/ethical committee approval was not required for this study, as it did not involve any patient data collection or impact on patient care. Experts were invited to participate by the ELBS ctDNA working group, participation was voluntary and there was no financial compensation for participation.

### Statistical analysis

Questionnaire results are summarized and displayed in a descriptive manner. Formal statistical analyses were not performed.

### Role of funders

This study was funded by the EU project CAN.HEAL, grant n.101080009 (KP, SAJ, PG). The funders had no role in the design, data collection, data analysis, interpretation, and/or writing of the article. The views and opinions expressed are those of the authors only and do not necessarily reflect those of the European Union or the European Commission. Neither the European Union nor the European Commission can be held responsible for them.

## Results

**Fig. 1:**
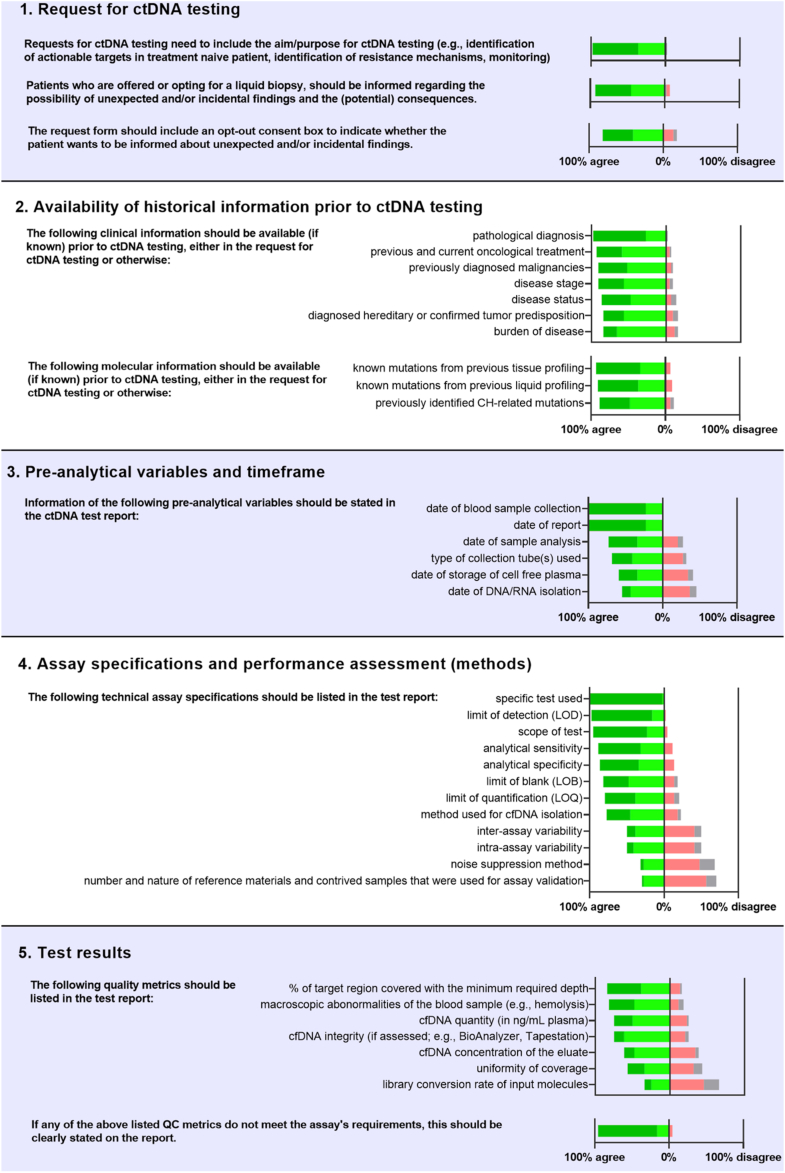

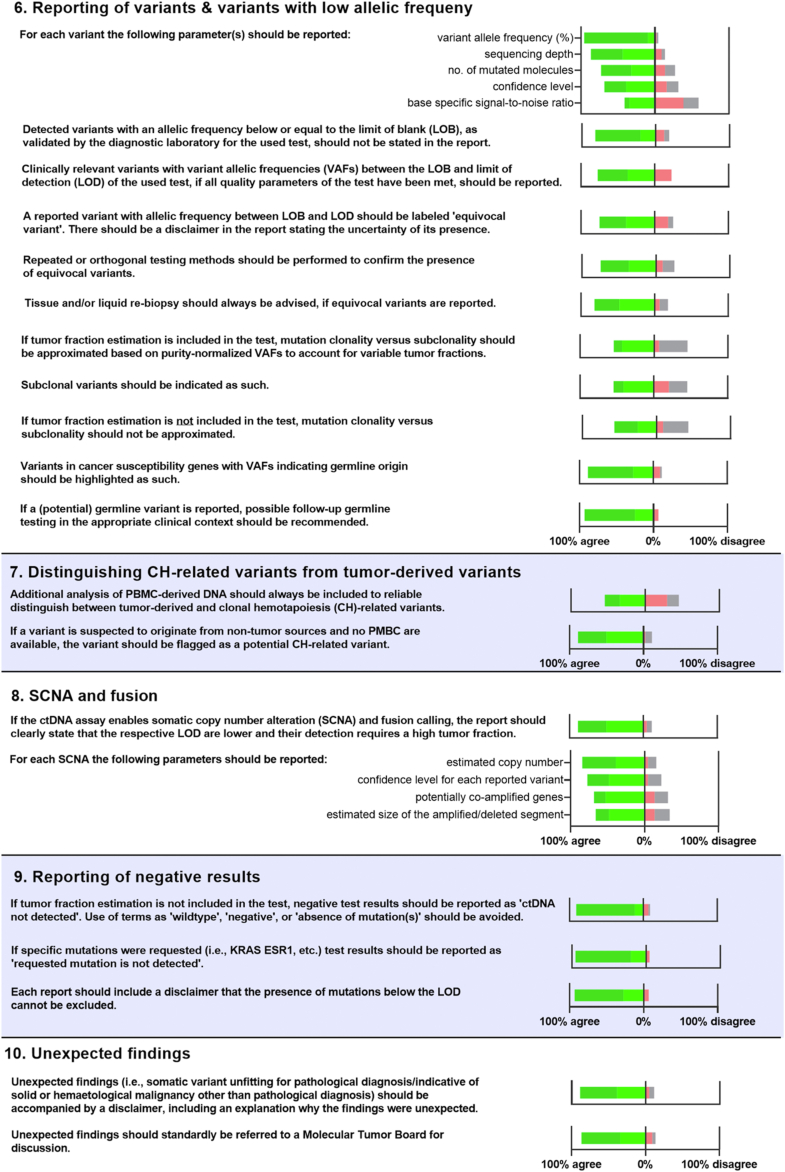
Overview of questionnaire answers. For each statement (in bold), experts provided their level of agreement for ctDNA test reporting. On the right of each statement, the distribution of all expert answers is depicted. Dark green represents ‘agree, essential’, light green represents ‘agree, useful’, red represents ‘disagree’, and grey represents ‘no opinion’.

All forty-four workshop attendees completed the questionnaire (response rate: 100%). The distribution of expert agreement levels is presented in Fig. 1. The ELBS expert opinion consensus recommendations for ctDNA reporting are presented in Table 2. A list of all statements and voting results is presented in Supplementary File S2.

### Request for ctDNA testing

There was consensus among experts regarding the upfront requirements for ctDNA testing. Firstly, requests for ctDNA testing need to include the aim/purpose, such as identification of resistance mechanisms. Secondly, patients should be informed regarding the possibility of unexpected/incidental findings, which refer to findings that were not the primary aim of the predictive diagnostic test, including germline variants and mutations associated with other cancer types. Germline mutations may suggest a predisposition to cancer that affects not only the patient but also their relatives, therefore genetic counselling and family screening should be recommended. Lastly, a ctDNA test request form should include an opt-out consent box to indicate whether a patient wishes to be informed about unexpected/incidental findings.

### Availability of historical information prior to ctDNA testing

The mutational profile of a tissue biopsy must be interpreted in the context of different clinical pathological variables, such as the type of malignancy, disease stage, pattern of metastatic disease, site of biopsy, presence of germline pathogenic variants, and prior lines of systemic treatment. The genomic profile of the total plasma ccfDNA is also affected by several elements, including the above. In addition, compared to a tumor specimen, ctDNA is affected by other variables such as the DNA-shedding source(s)—including the primary tumor of interest and its metastases, other undetected primary malignancies or metastases, as well as the hematopoietic cell background and clonality (CH, clonal hematopoiesis)—but also infections, tissue damage (vigorous exercise) and other comorbidities such as diabetes mellitus, cardiovascular disease and sepsis.^19^ Moreover, previous and current oncological treatment can greatly affect ctDNA levels. Since knowledge of a patient's history may help interpreting a molecular profiling result, the ELBS experts recommended that the request always mentions the purpose of the test and provides patient information with respect to (1) clinical and (2) molecular details (Table 2).

#### Clinical information

Technical limitations notwithstanding, CGP is expected to detect virtually any genomic variant released in plasma-derived ccfDNA, and not just somatic alterations released by the tumor. Incorrect labeling of these variants as tumor-specific of the oncological disease for which liquid biopsy testing is performed must be avoided.^20^ Moreover, though ctDNA is generally thought to be shed by all tumor sites, there are indications that the main contributors are the most aggressive lesions.^21^, ^22^, ^23^ Levels of ctDNA are often drastically influenced by the timing and response to anti-cancer therapy.^24^^,^^25^ While higher ctDNA levels are expected in a sample collected at baseline or at the time of disease progression, ctDNA levels are usually lower or even undetectable during (durable) treatment response. Lastly, healthcare professionals need to be aware that even in patients with high burden of disease, low or even undetectable levels of ctDNA may be observed. The underlying biological mechanisms for this variability are still largely unknown, but may be related to impairments in vascularization.^26^

Higher or lower ctDNA fractions may have different clinical validity depending on the extent of ccfDNA release. Information on tumor burden and organs affected by metastases in addition to tumor type and disease stage may aid interpretations, and should be succinctly described in the request for ctDNA testing (e.g., ‘non-small cell lung cancer, stage IV, metastatic disease limited to multiple cerebral lesions). This may help the diagnostic laboratory in defining the interpretation context of the molecular test results. If available, the following information should be provided with the test request form: pathological diagnosis, previous and current oncological treatment, previously diagnosed malignancies, disease stage, disease status, diagnosed hereditary or confirmed tumor predisposition, and burden of disease (see Fig. 1 and Supplementary File S2).

#### Molecular information

If previous molecular reports exist, the following information should be provided (if known) prior to ctDNA testing: known mutations from previous tissue profiling, known mutations from previous liquid profiling, and putative clonal hematopoiesis (CH)-related variants previously identified.

### Assay specifications and performance assessment

As with tissue-based testing, information on test type and scope needs to be incorporated into the ctDNA report. Control metrics that are used to assess the quality of NGS runs, such as gene coverage, sequencing depth and limit of detection (LoD) should be stated in the methodology section of the report. If any of the quality control metrics were not met, this should be stated in the conclusion and a warning should be added for the requesting physician. Moreover, any deviation from the standard operating protocol should be stated in the report.

The following technical assay specifications should be listed in the test report: specific test used, scope of the test, limit of detection (LoD), limit of blank (LoB), limit of quantification (LoQ), analytical sensitivity, analytical specificity, and method used for ccfDNA isolation. Reporting of inter-assay variability, intra-assay variability, noise suppression method and number and nature of reference materials and contrived samples used for assay validation were deemed less important (see Fig. 1 and Supplementary File S2).

### Quality metrics of test run

The probability to detect a genetic alteration is dependent on various factors, including the number of molecules assayed, the library conversion rate, the sequencing depth and the analytical sensitivity of a test. Despite substantial improvements in sequencing technologies and library preparation workflows, sequence errors may occur. In some cases, they give rise to signals comparable to (or higher than) the specific signals contributed by the mutations truly present in the test sample. Sequencing errors are a major challenge particularly when attempting to distinguish variants at low allelic fractions from background noise.

**Fig. 2:**
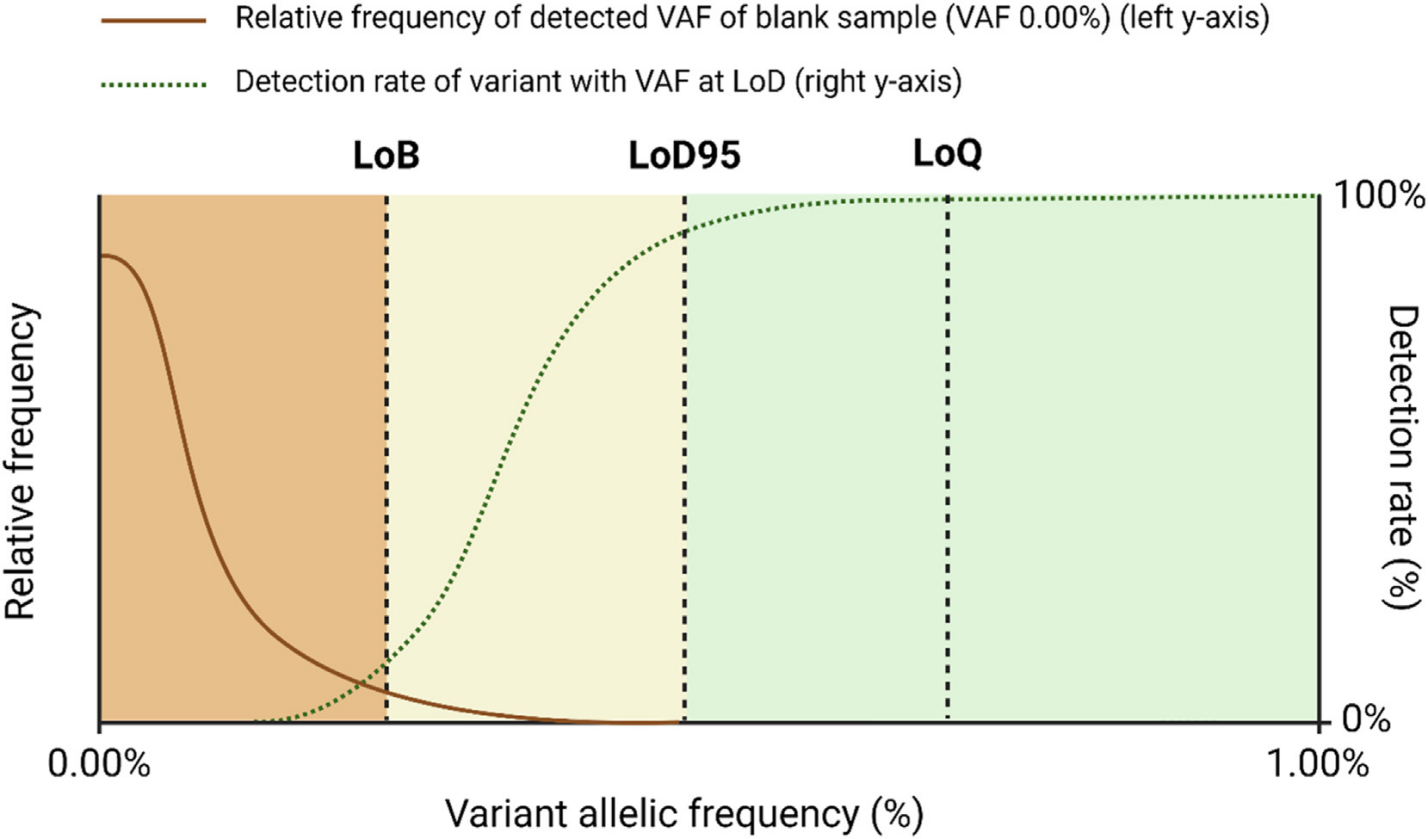
Relationship between the limit of blank (LoB), the limit of detection (LoD), and the limit of quantification (LoQ). The solid brown line represents the range of VAFs from blank sample(s), which are used to determine the LoB, which defines the background noise or the highest VAF expected from a control sample. The dotted green line represents the expected distribution of detections rates from positive samples with various VAFs at the LoD95, which defines the lowest VAF that can be reliably detected with 95% confidence. The LoQ is the lowest VAF of a variant that can be both detected and quantified with acceptable precision and accuracy. This means that not only can the analyte be detected, but the assay can also provide a reliable and reproducible measurement of its concentration. Adapted from Armbruster and Pry (2008).^27^ Created with www.BioRender.com.

The analytical sensitivity of a test is usually reported as the LoD95, which for ctDNA assays corresponds to the lowest quantity of ctDNA (either mutated copies or variant allele frequency, VAF) at which 95% of measurements will yield a positive result (see Fig. 2).^28^ Assay providers mostly calculate the LOD for optimized input levels of ccfDNA and a lower number of input molecules can negatively affect the detection probability.^29^

From the perspective of assay accuracy and reproducibility, variants reported above the LoD95 have a high (≥95%) confidence. Any variant reported below the LoD95 of the assay has less confidence, but can still be a true variant, given that the variant can be distinguished from noise signal measured in control samples (LoB, limit of blank).^28^^,^^30^ Since the signal-to-noise ratio can depend on the sequence context of the DNA, each detected variant is ideally reported with a confidence level considering the LoB for the respective base position.^31^

The following information with regard to test run quality should be listed in the report: percentage of target region covered with the minimum required depth and any macroscopic abnormalities of the blood sample (e.g., hemolysis). No consensus was reached for the reporting of ccfDNA concentration of the eluate, ccfDNA quantity (in ng/mL plasma), ccfDNA integrity, library conversion rate of input molecules and uniformity of coverage (see Fig. 1 and Supplementary File S2).

Importantly, if any of the quality metrics of the assay requirements are not met, this should be clearly stated on the report.

### Reporting of variants

Variant interpretation involves assessing both pathogenicity (the biological impact of the alteration in the oncogenesis process) and actionability (the clinical impact on therapy assignment). Variant interpretation bears implications on theranostic applications, as well as diagnostic and prognostic aspects (e.g., variant-driven sub-stratification of patients). Pathogenicity must be reported separately and prior to interpretation of actionability to avoid confusion.^32^, ^33^, ^34^ Among experts, the majority indicated that in the main text of the report only pathogenic and likely pathogenic variants should be included (36 [82%] of 44 experts), either with variants of unknown significance (VUS) reported as an Appendix (20 experts), with VUS and (likely) benign variants reported as an Appendix (8 experts), or without reporting of VUS or (likely) benign variants entirely (8 experts) (Supplementary File S2). Only eight [18%] experts indicated that pathogenic, likely pathogenic as well as VUS should all be included in the main report, while none of the experts would recommend including benign and likely benign variants.

In addition, for each variant the following parameter(s) should be reported: variant allele frequency, sequencing depth, number of mutated molecules per mL, and confidence level. No consensus was reached for the reporting of base specific signal-to-noise ratio (see Fig. 1 and Supplementary File S2).

#### Variant allele frequency below LoB

The LoB determines the point beyond which a signal can be considered above the noise. Therefore, variants within the range of LoB or below (orange area in Fig. 2) should not be listed in the report.

#### Variant allele frequency between LoB and LoD

Any measurement above the LoB but below the LoD95 has a high likelihood of being true, but could still be false positive noise (yellow area in Fig. 2). Moreover, VAFs below the LoQ might be detectable (i.e., above the LoD95) but not accurately quantifiable. From a clinical perspective, however, such findings may be relevant if the variant is actionable, which may warrant additional molecular testing at a later time point or with different clinical samples (i.e., tumor tissue). Importantly, previous studies have described effective targeted therapy despite VAF ≤0.2%.^35^, ^36^, ^37^ As of now, no consensus was reached for the reporting of clinically relevant variants with VAFs between the LoB and LoD (see Fig. 1 and Supplementary File S2). However, if such a variant is reported, it should be labeled ‘equivocal variant’, and there should be a disclaimer in the report stating the uncertainty of its presence. If possible, repeated and/or orthogonal testing methods should be performed to confirm the presence of equivocal variants. In this case, tissue and/or liquid re-biopsy should be advised. No consensus was reached for classifying a subclonal variant as such in the report (see Supplementary File S2).

### Distinguishing CH-related variants from tumor-derived variants

In addition to technical issues, calling for low frequency variant is further impaired by biological noise coming from clonal expansion of hematopoietic stem cells (CH, clonal hematopoiesis). CH refers to any clonal outgrowth of hematopoietic cells, regardless of cause or disease state, while the term ‘clonal hematopoiesis of indeterminate potential’ (CHIP) is used to indicate the presence of a haematological driver mutation (e.g., *DNMT3A*, *TET2*, or *ASXL1*) at a VAF of at least 2%.^38^, ^39^, ^40^ Mutations in genes commonly mutated in haematological malignancies, such as *JAK2, PPM1D, TP53, IDH2, SF3B1* and *SRSF2* have also been associated with CH.

CH has mainly been associated with age, but also with cytotoxic therapies such as chemo- and radiotherapy.^41^^,^^42^ As most of the ccfDNA originates from hematopoietic cells, CH-related mutations can be detected in a liquid biopsy and substantially contribute to the mutational profiles found in patients with cancer.^43^, ^44^, ^45^, ^46^, ^47^ Numerous ccfDNA studies have demonstrated that besides the hematology-related mutations, CH-mutations can also occur in genes that are frequently mutated in solid tumors, including *KRAS*, *GNAS*, *NRAS*, *PIK3CA* and many more, albeit mostly with VAF below 1%.^39^^,^^45^^,^^48^, ^49^, ^50^ Although such variants are more frequently originating from non-hematopoietic tumors, misinterpretations remain possible and may potentially lead to incorrect conclusions regarding the optimal treatment, prognosis or diagnosis.

Currently the only means to truly distinguish CH-related variants from tumor-derived variants in ccfDNA is to compare the plasma-derived ccfDNA with DNA from peripheral blood mononuclear cells (PBMC).^39^^,^^43^^,^^45^ However, for many diagnostic laboratories, systematic paired sequencing of ccfDNA and PBMC may not be feasible due to throughput and cost issues and no consensus was reached for the routine analysis of PBMC-derived DNA (see Fig. 1 and Supplementary File S2). Therefore, if a variant is suspected to originate from the hematopoietic compartment (Table 3) and PBMC testing is not performed, the variant should be flagged as a potential CH-related variant.

**Table 3:** Key situations when a variant in cfDNA is suspected to be of hematopoietic origin.

**Variant in common CHIP-associated genes**
Variants in certain genes are commonly associated with CHIP and are more likely to be of hematopoietic origin. Some of the most frequently mutated genes in CHIP include *DNMT3A, TET2, ASXL1, JAK2, TP53, SF3B1*. CHIP variants often show a low to intermediate VAF (usually 2–10%), reflecting the presence of the mutation in a subpopulation of blood cells.
**Low variant allele frequency**
The variant allele frequency (VAF) can be a clue. CH-related variants in non-canonical CHIP-gene are mostly below a VAF of 1%. In contrast, tumor-derived variants in cfDNA can have highly variable VAFs, depending on tumor burden, shedding rates, and other factors. Low VAF variants in samples with high ctDNA fractions may indicate that the variant is coming from hematopoietic cells rather than a tumor.
**Patient age**
CH is an age-related phenomenon, occurring significantly more frequently with increasing age. Therefore, in patients >50 years of age, especially in the absence of a known haematologic malignancy, somatic mutations detected in cfDNA may be CHIP-related.
**Absence of cancer-specific mutational profile**
Hematopoietic-origin variants typically do not follow the mutational patterns associated with the specific tumor type being analysed. For example, if a variant detected in cfDNA does not align with the typical mutation spectrum of a particular cancer (e.g., no typical driver mutations for that tumor type), it raises suspicion of a hematopoietic origin.
**Stability over time**
CH-associated mutations tend to be stable over time, as they represent clonal expansions of blood cells and are not subject to rapid evolutionary pressures like tumor mutations. If a cfDNA variant persists consistently over time without changes in VAF, and without corresponding changes in tumor markers or clinical progression, this may suggest a hematopoietic origin.
**Normal imaging and clinical findings**
If variants are found in cfDNA but there is no clinical evidence of active cancer (e.g., normal imaging, stable or low tumor markers), this increases suspicion that the detected variants may be from CH rather than tumor-derived.
**Confirmed haematologic findings**
If a patient has a known haematologic condition, such as a myelodysplastic syndrome (MDS) or other haematologic malignancy, variants detected in cfDNA could be from the hematopoietic system rather than from a solid tumor.

CHIP, clonal hematopoiesis of indeterminate potential.

### Somatic copy number alterations and fusions

In addition to genetic alterations at the nucleotide level, somatic copy number alterations (SCNA) and fusions may also occur as tumor drivers and are important predictive markers that can be identified in ccfDNA. In most clinical-grade NGS gene panels, SCNAs are called using a comparative genomic hybridization-like method, in which a log–ratio profile of the sample is obtained by normalizing the sequence coverage obtained at the target region against a process-matched normal control. Log-ratios are segmented and interpreted using VAF of single nucleotide variants (SNVs) to estimate tumor purity and copy number at each segment. However, low ctDNA fractions may limit accuracy, particularly for SCNA and fusion calling.^51^^,^^52^ Therefore, if SCNA and fusion calling is included in the ctDNA assay, the report should clearly state that the respective LoDs are lower and their detection requires a high tumor fraction.

Another (still unexplored) challenge is mosaicism of copy number variants (CNV) that could potentially be identified as SCNA. In order to reconstruct genome-wide copy number profile a tumor fraction of around 5% is needed, while highly amplified regions may also be detected in samples with ∼1% of tumor content.^53^, ^54^, ^55^

For each SCNA, the following parameters should be reported: estimated copy number, estimated size of the amplified/deleted segment, potentially co-amplified genesand confidence level (see Fig. 1 and Supplementary File S2).

### Tumor mutational burden

Tumor mutational burden (TMB) denotes a relative number of somatic mutations in a tumor genome.^56^ Most often, TMB is expressed as the number of somatic mutations per megabase (mut/Mb) of DNA. Previous studies have demonstrated, both retro- and prospectively, that tissue-based TMB carries predictive value for treatment with mono-immunotherapy in many types of solid malignancies.^56^ Based on the results of the phase II KEYNOTE-158 trial, the Food and Drug Administration (FDA) approved the use of pembrolizumab, a PD-1 inhibitor, in patients with an unresectable or metastatic solid tumor with high tissue-based TMB (defined as >10 mut/Mb, as determined by an FDA-approved test).^57^ The main challenges in the implementation of tissue-based TMB (tTMB) as a routine biomarker for immunotherapy treatment include the requirement for a sufficient tissue quantity, the heterogeneity in the approaches for determining TMB and the variation in TMB cut-off scoring.^58^ Using ctDNA testing to measure TMB eliminates the need for sufficient tumor tissue. Several studies have retrospectively demonstrated the putative predictive value of blood-based TMB (bTMB) for clinical benefit of immunotherapy in patients with non-small cell lung cancer.^59^ However, in other observational studies the utility of bTMB to predict immunotherapy outcome was more limited (e.g., dependent on changes in bTMB and not their absolute levels).^60^ Moreover, the use of bTMB to select patients for immunotherapy has not yet resulted in the anticipated benefit in prospective trials (e.g., phase III BFAST trial).^61^ In conclusion, tTMB is a promising biomarker for patient selection for immunotherapy and monitoring of response to immunotherapy and has been introduced as a routine biomarker in some countries. Nevertheless, optimal use of tTMB is not well-established and data on its usefulness is limited in many tumor types. Using bTMB as an alternative to tTMB is predominantly limited to (translational) research. Therefore, bTMB was not (yet) included in the recommended metrics in routine liquid biopsy reports during the ELBS ctDNA workshop.

### Reporting of negative results

Reporting test results of ctDNA-based NGS when no (pathogenic) variants are identified should be done with caution, as these findings can be false negative results due to technical issues, insufficient amounts or poor quality of cfDNA and/or insufficient sensitivity of the ctDNA test performed. If tumor fraction estimation is not included in the test, negative test results should be reported as ‘ctDNA not detected’. As recommended previously, use of terms as ‘wildtype’, ‘negative’, or ‘absence of mutation(s)’ should be avoided.^11^^,^^12^^,^^14^^,^^15^ If testing of specific mutations was requested (i.e., *KRAS*, *ESR1*, etc.), results should be reported as ‘requested mutation is not detected’. It is important for clinical molecular biologists and clinicians to be aware that ctDNA-based testing may produce false-negative results for low-shedding tumors. For instance, the detection of clinically relevant variants in lung cancer comparing both tumor tissue and matched plasma ccfDNA reveals a sensitivity of 70–75%,^62^^,^^63^ illustrating a false-negative rate of at least 30%. For this reason, each report should include a disclaimer that the presence of mutations below the LOD cannot be excluded.

### Actionability

Once a variant is classified as pathogenic or likely pathogenic, its actionability depends on the clinical context. Two major factors to be considered are cancer histology and disease status (e.g., early cancer, loco-regional relapse, metastatic spread, response to therapy).^34^ Since a pathogenic variant may be actionable in one cancer type but not in another or actionability may be supported at different levels of evidence in different histological or molecular subtypes. As a result, it is still a matter of debate whether or not a uniform threshold of actionability should be enforced and mentioned in diagnostic reports of actionability. The majority of experts indicated that actionability should be reported for variants with tier I/II evidence according to the ESMO Scale of Clinical Actionability for molecular Targets (ESCAT) (21 [48%] of 44 experts, Supplementary File S2). Similar but not identical opinion was expressed by 7 [16%] of 44 experts who recommended reporting actionability only for ‘unequivocally targetable alterations’ without explicit mention of the scale of actionability. Other experts indicated that clinical annotations should only be done by a Molecular Tumor Board (MTB) (9 [20%] of 44 experts) or that treatment recommendations should never be included in the report (7 [16%] of 44 experts). Since no complete agreement was reached among experts, we recommend to disclose clinically actionable results and evidence-based associations with drug responses, but patient-specific, individual treatment recommendations should not be given.^13^ The actual match between clinical annotation and treatment for a specific variant should remain with the clinical oncologist, the institutional organ-specific disease-treatment board, and/or the MTB.

### Unexpected findings

From the perspective of test reporting, unexpected findings of liquid biopsy tests should be accompanied by a disclaimer/warning, including an explanation why the findings were unexpected. It is recommended that unexpected findings are standardly discussed in an MTB.

Strong evidence for the effectiveness of MTB-recommended treatment is limited due to the heterogeneity of patients accrued and the complexity of outcome assessment in this setting, as well as the variation in MTB definition and functioning.^64^ Nevertheless, previous studies have described treatment outcomes of patients discussed in MTBs that support their putative beneficial role.^64^, ^65^, ^66^ The workshop experts acknowledge the diversity of the (national) legislations applicable to medical laboratories and institutions. As such, involved authorities and the options to provide (treatment) recommendations may differ. However, if the option exists to discuss ctDNA test results in a regional or institutional MTB, it is recommended that this possibility is included as a reminder in the test report.

**Fig. 3:**
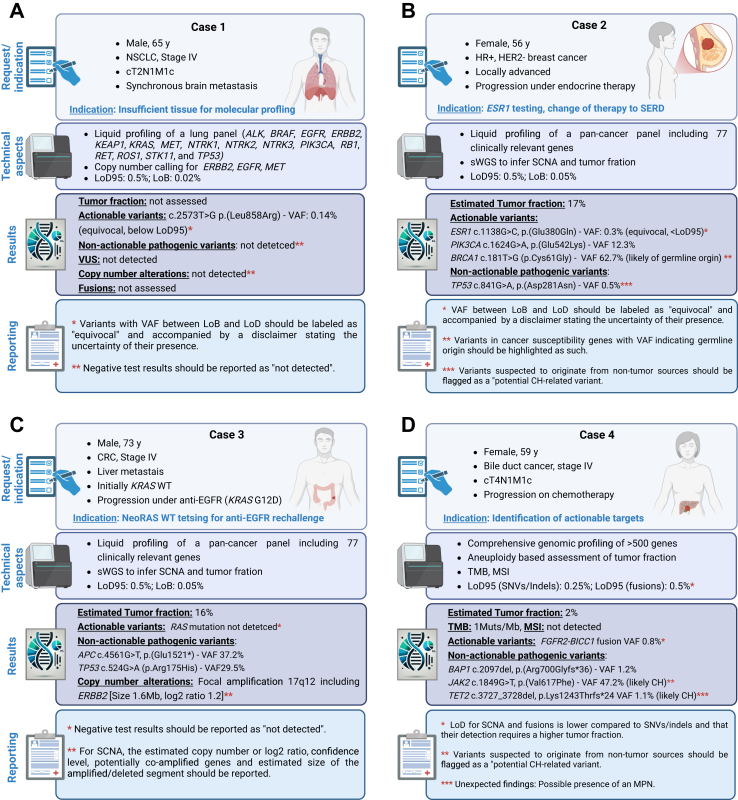
Four examples (A to D) of a clinical case involving a request for plasma-based predictive molecular profiling. Each example includes a typical molecular result, an interpretation of the NGS findings referencing the ELBS recommendations for reporting, and a sample report (Supplementary Files S3, S4, S5 and S6).

In Fig. 3, we provide four distinct examples, each illustrating a clinical case involving a request for plasma-based predictive molecular profiling. Each example includes a typical molecular result, an interpretation of the NGS findings referencing the ELBS recommendations for reporting (where applicable), and a sample report (Supplementary Files S3–S6). These example report formats are designed to offer practical insights for oncologists, geneticists, and clinical molecular biologists. They can also serve as a foundational template for laboratories when adopting liquid biopsy testing and reporting of molecular findings.

## Conclusions

Predictive liquid biopsy biomarker reports must be technically accurate while ultimately prioritizing patient benefit. To achieve this, these reports should be seamlessly integrated into an optimized treatment decision-making workflow. Given the unique complexities of ctDNA testing, which introduces multiple layers of clinical-pathological intricacy not typically encountered in tumor tissue analysis, collaboration and communication among clinical scientists, (molecular) pathologists, geneticists, and oncologists are expected to intensify in the near future. To address the significant challenges of liquid biopsy result reporting, this report presents consensus recommendations for ctDNA test reporting informed by extensive real-world experience in the field (Table 2). In contrast to existing recommendations, a key strength of this effort is the transparency of the consensus-building process and the involvement of a broad panel of experts with real-world experience in liquid profiling. This report represents a valuable addition to existing guidelines, since it evaluates and prioritizes the significance of each recommendation based on the consensus of an expert panel offering a practical guide for laboratories worldwide. By facilitating the implementation of liquid biopsy testing as a routine tool in precision oncology, this guide aims to enhance communication between oncologists and diagnostic teams, ultimately improving patient care.

Practical responsibilities for (medical) oncologists requesting ctDNA testing include providing the testing laboratory with relevant clinical information and ensuring patients are appropriately informed about the benefits and limitations of ctDNA testing. This includes explicitly acknowledging the possibility of unexpected and incidental findings. For clinical scientists, (molecular) pathologists, and geneticists, the key challenges in ctDNA test reporting involve managing variants detected at low VAFs, potential clonal hematopoiesis (CH)-derived variants, somatic copy number alterations (SCNA), and fusions. These findings must be described adequately but concisely, with a synoptic explanation of their clinical relevance, ensuring clear and unequivocal communication with the treating physician and, ultimately, the patient.

Both oncologists and laboratory personnel need to be aware of challenges and potential uncertainties of test results. Clinical scientists (molecular) pathologists, and geneticists should support oncologists in interpreting ctDNA test reports, especially given that many oncologists may not yet be fully familiar with this complex information. Conversely, oncologists should invest in clarifying the intricacies of clinical cases to laboratory professionals, fostering continuous bilateral communication before, during, and after diagnostic reporting. This collaborative approach ensures tailored expertise for each clinical case and optimal patient management.

## Contributors

VdJ: data curation, formal analysis, investigation, methodology, visualization, writing–original draft, writing–review & editing. PG, SJ: conceptualization, funding acquisition, investigation, methodology, writing–originial draft, writing–review & editing. JF, RT, SP, ZD: conceptualization, investigation, methodology, writing–originial draft, writing–review & editing. CK: conceptualization, data curation, formal analysis, investigation, methodology, project administration, visualization, writing–original draft, writing–review & editing. EH, ES: conceptualization, data curation, formal analysis, investigation, methodology, supervision, visualization, writing–original draft, writing–review & editing. SA, CLA, DA, BB, IRB, DvdB, ED, BGP, AG, AH, JH, MH, SI, LK, MK, ML, LLC, MMV, KN, MN, BN, AOn, SO, AOs, NP, MHR, ER, AR, MR, HS, US, LSP, HS, PT, NW: investigation, writing–review & editing. All authors read and approved the final version of the manuscript.

## Data sharing statement

The questionnaire and the aggregated participant results are available in Supplementary File S2.

## Declaration of interests

VdJ has received speaker's fees from Roche and Janssen (Johnson & Johnson) (all paid to institution). PG has received payment or honoraria from Illumina for Round Tables on Comprehensive Genomic Profiling, and has a leadership or fiduciary role in an Advisory Board for Thena Biotech. RT has received grants or contracts from AstraZeneca, Beigene pharmaceutics, and Personalis. SP has received financial support from AstraZeneca, MSD, and Johnson & Johnson for EMQN CIC to deliver external quality assessment activities to laboratories worldwide, has received honoraria from AstraZeneca for delivering webinar series, and has received travel costs from AstraZeneca to support delivery of a lecture at a major European conference.SJ has received funding from the EU from the EU4Health call (project name: Building the EU Cancer and Public Health Genomics platform (CAN.HEAL). ES has received unrestricted grants (all paid to UMCG institution) from Abbott, Biocartis, AstraZeneca, Invitae/Archer, Bayer, Bio-Rad, Roche, Agena Bioscience, CC Diagnostics, MSD/MERCK, and SNN/EFRO, has received consulting fees (all paid to UMCG institution) from MSD/Merck, AstraZeneca, Roche, Novartis, Bayer, BMS, Lilly, Amgen, Illumina, Agena Bioscience, CC Diagnostics, Janssen Cilag (Johnson & Johnson), Astellas Pharma, GSK, Sinnovisionlab, Sysmex, and Protyon, has received payments or honoraria (all paid to UMCG institution) from Bio-Rad, Seracare, Roche, Biocartis, Lilly, Agena Bioscience, and Illumina, has received support for attending meetings and/or travel from BioRad, Biocartis, Ageno Sciences, Illumina, Roche/Foundation Medicine, and QCMD, is a board member for the Dutch Society of Pathology (unpaid), European Society of Pathology (unpaid), European Liquid Biopsy Society (unpaid), is a secretary/member of the advisory committee for assessment of molecular diagnostics (cieBOD) (honoraria paid to UMCG institution), is committee member of national guideline advisory (honoraria paid to UMCG institution). CLA has received support for the present manuscript from EU CAN.HEAL (grant n.101080009) (payment to institution). BB has received Oncomine Research Grant from ThermoFisher for analysis of cfDNA in SCLC. AG has received consulting fees from Guardant Health (personal payment) and Foundation Medicine (personal payment). TJNH has received grants or contracts from Roche, BMS, and AstraZeneca (all payments to institution). MH has received consulting fees from Guardant Health, AstraZeneca, Boehringer Ingelheim, Bayer, Roche, Novartis, Merck, Incyte, GSK, and Qiagen, has received payment or honoraria for lectures, presentations, speakers bureaus, manuscript writing or educational events from Janssen, Servier, Bayer, and Seagen, and has received support for attending meetings and/or travel from Janssen, Servier, Nayer, and Seagen. SI has received grants or contracts from Menarini Stemline (contract with own institution (UNIPD)), has received honorarium for one lecture from Sysmex Partec Srl, and has received support for attending meetings and/or travel from Roche Italy SpA (invitation to a Roche sponsored meeting). LK has received payment for expert testimony from AstraZeneca (payment to institution), and has received support for attending meetings and/or travel from AstraZeneca and Roche. MK has received institutional research grants from Roche and Novartis, has received advisory board/consultancy fees from Janssen, Roche, Bayer, Guardant Health, and Zai Lab, has received payment or honoraria for speakers bureaus from Janssen and Roche, and has received support for travel expenses from Janssen, Roche, and Zai Lab. ML has received grants or contracts from KWF Dutch cancer society (payments to institution), has received consulting fees from GlaxoSmithKline B.V. (payments to institution)and Janssen-Cilag B.V. (payments to institution), and AstraZeneca (payments to institution), and has received payments or honoraria for lectures, presentations, speakers bureaus, manuscript writing or educational events from AstraZeneca, Janssen, and Roche (payments to institution) and from AstraZeneca, Janssen, MSD, and Pfizer (payments to institution). MMV has received grants or contracts from AstraZeneca (research contract to own institution), Sumitomo (research contract to own institution), and Merck KGaA (research contract to own institution), and has received payment or honoraria for lectures, presentations, speakers' bureaus, manuscript writing or educational events from The Ricky Rubio Foundation and IX Spanish Symposium of Liquid Biopsy. KN has other financial or non-financial interests as employee of LGC Clinical Diagnostics. SO has received support for the present manuscript from EU project CAN.HEAL (grant n.101080009), German Research Foundation (DFG) DFG_GZ: OS647/18-1, and DFG DFG_GZ: OS647/14-1, has received grants or contracts from DFG DFG_GZ: OS 647/18-1 (research grant), DFG DFG_GZ: OS 647/14-1 (research grant), European Union's H2020: Can.Heal, European Union's H2020: Melcaya, DFG DFG_OS 647/13-1, DFG DFG_OS 647/7-1, Stiftung Deutsche Krebshilfe: HerediVar, Network of University Medicine for Covid-19 (NUM) GenSurv, and DFG DFG_OS 647/1-1 (all research grants), has received payment or honoraria from Illumina Inc. for presentation at GfH Conference, has received support for attending meetings and/or travel from Illumina Inc. for GfH Conference (travel, hotel, conference fees), and Oxford Nanopore Technologies (travel, hotel, conference fees), and has patents planned, issued or pending (PCT/EP2023/061,370, not related to ctDNA testing). AO has received payments or honoraria for lectures, presentations, speakers bureaus, manuscript writing or educational events from Illumina, Inc. and Stemline Therapeutics B.V., has received support for attending meetings and/or travel from Illumina, Inc., and has participated on a Data Safety Monitoring Board or Advisory Board for Illumina, Inc. ER has received grants or contracts from AstraZeneca, Roche Diagnostics, Clovis, GSK, and BMS, has received consulting fees from AstraZeneca, Roche Diagnostics, Clovis, GSK, and BMS, and has received support for attending meetings and/or travel from AZ and BMS. AR has received a Clinical Scientist Fellowship from Cancer Research UK, has received Horizon Europe Guarantee Funding (PANCAID) from United Kingdom Research and Innovation, has a leadership or fiduciary role as and has received honoraria as Director of Biology Research, ACED from Alliance for Cancer Early Detection, and has a leadership or fiduciary role in the National Research Committee of the British Association for Plastic and Reconstructive Surgery. HS has received institutional funding of German Cancer Research Center (DKFZ); competitive third-party funding through the German Centres for Lung Disease (DZL) and the German Cancer Consortium (DKTK) from the German Federal Ministry for Education and Research. All other authors reports no conflicts of interest.
