# Supplementary material for: Reporting of molecular test results from cell-free DNA analyses: expert consensus recommendations from the 2023 European Liquid Biopsy Society ctDNA Workshop
Source: eBioMedicine. 2025 Mar 22;114:105636. doi: 10.1016/j.ebiom.2025.105636 (PMC11979934; doi:10.1016/j.ebiom.2025.105636)
Supplement: Supplementary File S1 [file mmc1.docx]

**Supplementary File S1.** Questionnaire

1. What is your name?
2. What is your profession?
   1. (Pulmonary) oncologist
   2. (Molecular) pathologist
   3. Clinical scientist in molecular pathology
   4. Clinical laboratory geneticist
   5. Molecular biologist
   6. Researcher
   7. Other
3. Patients, who are offered or opting for a liquid biopsy, should be informed regarding the possibility of unexpected and/or incidental findings and the (potential) consequences.
   1. Agree, essential
   2. Agree, useful
   3. Disagree
   4. No opinion
4. If needed, you can explain your answer to the above statement(s) (referring to point 3):
5. The request form should include an opt-out consent box to indicate whether the patient wants to be informed about unexpected and/or indidental findings.
   1. Agree, essential
   2. Agree, useful
   3. Disagree
   4. No opinion
6. If needed, you can explain your answer to the above statement(s) (referring to point 5):
7. Requests for ctDNA testing need to include the aim/purpose for ctDNA testing (e.g., identification of actionable targets in treatment-naive patient, identification of resistance mechanisms, monitoring)
   1. Agree, essential
   2. Agree, useful
   3. Disagree
   4. No opinion
8. If needed, you can explain your answer to the above statement(s) (referring to point 7):
9. The following clinical information should be available (if known) prior to ctDNA testing, either in the request for ctDNA testing or otherwise:

9.1. pathological diagnosis

- 1. Agree, essential
  2. Agree, useful
  3. Disagree
  4. No opinion

9.2. disease stage

1. Agree, essential
2. Agree, useful
3. Disagree
4. No opinion

9.3. burden of disease (basic information on the number and localization of metastases)

1. Agree, essential
2. Agree, useful
3. Disagree
4. No opinion

9.4. disease status (PD, SD, PR, CR)

1. Agree, essential
2. Agree, useful
3. Disagree
4. No opinion

9.5. previous and current oncological treatment

1. Agree, essential
2. Agree, useful
3. Disagree
4. No opinion

9.6. previously diagnosed malignancies

1. Agree, essential
2. Agree, useful
3. Disagree
4. No opinion

9.7. diagnosed hereditary or confirmed tumor predisposition

1. Agree, essential
2. Agree, useful
3. Disagree
4. No opinion
5. If needed, you can explain your answer to the above statement(s) (referring to point 9):
6. The following molecular information should be available prior to ctDNA testing, either in the request for ctDNA testing or otherwise:

11.1. known mutations from previous tissue profiling

1. Agree, essential
2. Agree, useful
3. Disagree
4. No opinion

11.2. known mutations from previous liquid profiling

1. Agree, essential
2. Agree, useful
3. Disagree
4. No opinion

11.3. previously identified CH-related mutations

1. Agree, essential
2. Agree, useful
3. Disagree
4. No opinion
5. If needed, you can explain your answer to the above statement(s) (referring to point 11):
6. The following clinical information should be stated in the final ctDNA report:

13.1. pathological diagnosis

1. Agree, essential
2. Agree, useful
3. Disagree
4. No opinion

13.2. disease stage

1. Agree, essential
2. Agree, useful
3. Disagree
4. No opinion

13.3. burden of disease (basic information on the number and localization of metastases)

1. Agree, essential
2. Agree, useful
3. Disagree
4. No opinion

13.4. disease status (PD, SD, PR, CR)

1. Agree, essential
2. Agree, useful
3. Disagree
4. No opinion

13.5. previous and current oncological treatment

1. Agree, essential
2. Agree, useful
3. Disagree
4. No opinion

13.6. previously diagnosed malignancies

1. Agree, essential
2. Agree, useful
3. Disagree
4. No opinion

13.7. diagnosed hereditary or confirmed tumor predisposition

1. Agree, essential
2. Agree, useful
3. Disagree
4. No opinion
5. If needed, you can explain your answer to the above statement(s) (referring to point 13):
6. The following historical molecular information should be stated in the final ctDNA report:

15.1. known mutations from previous tissue profiling

1. Agree, essential
2. Agree, useful
3. Disagree
4. No opinion

15.2. known mutations from previous liquid profiling

1. Agree, essential
2. Agree, useful
3. Disagree
4. No opinion

15.3. previously identified CH-related mutations

1. Agree, essential
2. Agree, useful
3. Disagree
4. No opinion
5. If needed, you can explain your answer to the above statement(s) (referring to point 15):
6. Information of the following pre-analytical variables should be stated in the ctDNA test report:

17.1. type of collection tube(s) used

1. Agree, essential
2. Agree, useful
3. Disagree
4. No opinion

17.2. date of blood sample collection

1. Agree, essential
2. Agree, useful
3. Disagree
4. No opinion

17.3. date of storage of cell free plasma

1. Agree, essential
2. Agree, useful
3. Disagree
4. No opinion

17.4. date of DNA/RNA isolation

1. Agree, essential
2. Agree, useful
3. Disagree
4. No opinion

17.5. date of sample analysis

1. Agree, essential
2. Agree, useful
3. Disagree
4. No opinion

17.6. date of report

1. Agree, essential
2. Agree, useful
3. Disagree
4. No opinion
5. If needed, you can explain your answer to the above statement(s) (referring to point 17):
6. The following technical assay specifications should be listed in the test report:

19.1. specific test used

1. Agree, essential
2. Agree, useful
3. Disagree
4. No opinion

19.2. scope of test

1. Agree, essential
2. Agree, useful
3. Disagree
4. No opinion

19.3. method used for cfDNA isolation

1. Agree, essential
2. Agree, useful
3. Disagree
4. No opinion

19.4. limit of detection (LOD)

1. Agree, essential
2. Agree, useful
3. Disagree
4. No opinion

19.5. limit of quantification (LOQ)

1. Agree, essential
2. Agree, useful
3. Disagree
4. No opinion

19.6. limit of blank (LOB)

1. Agree, essential
2. Agree, useful
3. Disagree
4. No opinion

19.7. analytical sensitivity

1. Agree, essential
2. Agree, useful
3. Disagree
4. No opinion

19.8. analytical specificity

1. Agree, essential
2. Agree, useful
3. Disagree
4. No opinion

19.9. inter-assay variability

1. Agree, essential
2. Agree, useful
3. Disagree
4. No opinion

19.10. intra-assay variability

1. Agree, essential
2. Agree, useful
3. Disagree
4. No opinion

19.11. noise suppression method

1. Agree, essential
2. Agree, useful
3. Disagree
4. No opinion

19.12. number and nature of reference materials and contrived samples that were used for assay validation

1. Agree, essential
2. Agree, useful
3. Disagree
4. No opinion
5. If needed, you can explain your answer to the above statement(s) (referring to point 19):
6. The following quality metrics should be listed in the test report:

21.1. macroscopic abnormalities of the blood sample (e.g., hemolysis)

1. Agree, essential
2. Agree, useful
3. Disagree
4. No opinion

21.2. cfDNA concentration of the eluate

1. Agree, essential
2. Agree, useful
3. Disagree
4. No opinion

21.3. cfDNA quantity (in ng/mL plasma)

1. Agree, essential
2. Agree, useful
3. Disagree
4. No opinion

21.4. cfDNA integrity (if assessed; e.g., BioAnalyzer, Tapestation)

1. Agree, essential
2. Agree, useful
3. Disagree
4. No opinion

21.5. library conversion rate of input molecules

1. Agree, essential
2. Agree, useful
3. Disagree
4. No opinion

21.6. uniformity of coverage

1. Agree, essential
2. Agree, useful
3. Disagree
4. No opinion

21.7. % of target region covered with the minimum required depth

1. Agree, essential
2. Agree, useful
3. Disagree
4. No opinion
5. If needed, you can explain your answer to the above statement(s) (referring to point 21):
6. If any of the above listed QC metrics (referring to point 21) do not meet the assay's requirements, this should be clearly stated on the report.
   1. Agree, essential
   2. Agree, useful
   3. Disagree
   4. No opinion
7. If needed, you can explain your answer to the above statement(s) (referring to point 23):
8. For each variant the following parameter(s) should be reported:

25.1. variant allele frequency (%)

25.2. no. of mutated molecules

25.3. sequencing depth

25.4. base specific signal-to-noise ratio

25.5. confidence level

1. If needed, you can explain your answer to the above statement(s) (referring to point 25):
2. Please read all below statements regarding reporting of variants and choose one of the options.
   1. Only pathogenic and likely pathogenic variants should be included in the report.
   2. Only pathogenic variants, likely variant and VUS should be included in the report.
   3. Only pathogenic and likely pathogenic variants should be included in the main report. VUS should be listed as appendix.
   4. Only pathohenic and likely pathogenic variants should be included in the main report. Benign variants, likely benign variants and variants of unknown significance (VUS) should be listed as appendix.
   5. All variants, including benign and likely benign variants, should be included in the report.
3. If needed, you can explain your answer to the above statement(s) (referring to point 27):
4. Detected variants with an allelic frequency below or equal to the limit of blank (LOB), as validated by the diagnostic laboratory for the used test, should not be stated in the report.
   1. Agree, essential
   2. Agree, useful
   3. Disagree
   4. No opinion
5. If needed, you can explain your answer to the above statement(s) (referring to point 29):
6. Clinically relevant variants with variant allelic frequencies (VAFs) between the LOB and limit of detection (LOD) of the used test, if all quality parameters of the test have been met, should be reported.
   1. Agree, essential
   2. Agree, useful
   3. Disagree
   4. No opinion
7. If needed, you can explain your answer to the above statement(s) (referring to point 31):
8. A reported variant with allelic frequency between LOB and LOD should be labeled 'equivocal variant'. There should be a disclaimer in the report stating the uncertainty of its presence.
   1. Agree, essential
   2. Agree, useful
   3. Disagree
   4. No opinion
9. If needed, you can explain your answer to the above statement(s) (referring to point 33):
10. Repeated or orthogonal testing methods should be performed to confirm the presence of equivocal variants.
    1. Agree, essential
    2. Agree, useful
    3. Disagree
    4. No opinion
11. If needed, you can explain your answer to the above statement(s) (referring to point 35):
12. Tissue and/or liquid re-biopsy should always be advised, if equivocal variants are reported.
    1. Agree, essential
    2. Agree, useful
    3. Disagree
    4. No opinion
13. If needed, you can explain your answer to the above statement(s) (referring to point 37):
14. If tumor fraction estimation is included in the test, mutation clonality versus subclonality should be approximated based on purity-normalized VAFs to account for variable tumor fractions.
    1. Agree, essential
    2. Agree, useful
    3. Disagree
    4. No opinion
15. If needed, you can explain your answer to the above statement(s) (referring to point 39):
16. Subclonal variants should be indicated as such.
    1. Agree, essential
    2. Agree, useful
    3. Disagree
    4. No opinion
17. If needed, you can explain your answer to the above statement(s) (referring to point 41):
18. If tumor fraction estimation is not included in the test, mutation clonality versus subclonality should not be approximated.
    1. Agree, essential
    2. Agree, useful
    3. Disagree
    4. No opinion
19. If needed, you can explain your answer to the above statement(s) (referring to point 43):
20. Variants in cancer susceptibility genes with VAFs indicating germline origin should be highlighted as such.
    1. Agree, essential
    2. Agree, useful
    3. Disagree
    4. No opinion
21. If needed, you can explain your answer to the above statement(s) (referring to point 45):
22. If a (potential) germline variant is reported, possible follow-up germline testing in the appropriate clinical context should be recommended.
    1. Agree, essential
    2. Agree, useful
    3. Disagree
    4. No opinion
23. If needed, you can explain your answer to the above statement(s) (referring to point 47):
24. Additional analysis of PBMC-derived DNA should always be included to reliable distinguish between tumor-derived and clonal hemotapoiesis (CH)-related variants.
    1. Agree, essential
    2. Agree, useful
    3. Disagree
    4. No opinion
25. If needed, you can explain your answer to the above statement(s) (referring to point 49):
26. If a variant is suspected to originate from non-tumor sources and no PMBC are available, the variant should be flagged as a potential CH-related variant.
    1. Agree, essential
    2. Agree, useful
    3. Disagree
    4. No opinion
27. If needed, you can explain your answer to the above statement(s) (referring to point 51):
28. If the ctDNA assay enables somatic copy number alteration (SCNA) and fusion calling, the report should clearly state that the respective LOD are lower and their detection requires a high tumor fraction.
29. If needed, you can explain your answer to the above statement(s) (referring to point 53):
30. For each SCNA the following parameters should be reported:

55.1. estimated copy number

- 1. Agree, essential
  2. Agree, useful
  3. Disagree
  4. No opinion

55.2. estimated size of the amplified/deleted segment

1. Agree, essential
2. Agree, useful
3. Disagree
4. No opinion

55.3. potentially co-amplified genes

1. Agree, essential
2. Agree, useful
3. Disagree
4. No opinion

55.4. confidence level for each reported variant

1. Agree, essential
2. Agree, useful
3. Disagree
4. No opinion
5. If needed, you can explain your answer to the above statement(s) (referring to point 55):
6. If tumor fraction estimation is not included in the test, negative test results should be reported as 'ctDNA not detected'. Use of terms as 'wildtype', 'negative', or 'absence of mutation(s)' should be avoided.
   1. Agree, essential
   2. Agree, useful
   3. Disagree
   4. No opinion
7. If needed, you can explain your answer to the above statement(s) (referring to point 57):
8. If specific mutations were requested (i.e., KRAS ESR1, etc.) test results should be reported as 'requested mutation is not detected'.
   1. Agree, essential
   2. Agree, useful
   3. Disagree
   4. No opinion
9. If needed, you can explain your answer to the above statement(s) (referring to point 59):
10. Each report should include a disclaimer that the presence of mutations below the LOD cannot be excluded.
11. If needed, you can explain your answer to the above statement(s) (referring to point 61):
12. Unexpected findings (i.e., somatic variant unfitting for pathological diagnosis/indicative of solid or hemaetological malignancy other than pathological diagnosis) should be accompanied by a disclaimer, including an explanation why the findings were unexpected.
    1. Agree, essential
    2. Agree, useful
    3. Disagree
    4. No opinion
13. If needed, you can explain your answer to the above statement(s) (referring to point 63):
14. Unexpected findings should standardly be referred to a Molecular Tumor Board for discussion.
    1. Agree, essential
    2. Agree, useful
    3. Disagree
    4. No opinion
15. If needed, you can explain your answer to the above statement(s) (referring to point 65):
16. Please read all below statements regarding reporting of variants and choose one of the options.
    1. Variant should only be matched with potential therapies in the report if evidence-based treatment recommendations (tier 1, ESCAT 1 or 2) are available.
    2. Treatment recommendations should only be listed for unequivocally actionable alterations.
    3. Treatment recommendations should never be included in the report.
    4. Clinical annotations should only be done by an MTB
17. If needed, you can explain your answer to the above statement(s) (referring to point 67):
