# Supplementary material for: Reporting of molecular test results from cell-free DNA analyses: expert consensus recommendations from the 2023 European Liquid Biopsy Society ctDNA Workshop
Source: eBioMedicine. 2025 Mar 22;114:105636. doi: 10.1016/j.ebiom.2025.105636 (PMC11979934; doi:10.1016/j.ebiom.2025.105636)
Supplement: Supplementary File S2 [file mmc2.docx]

**Supplementary File S2.**

| **Overview of answers to questionnaire with statements for ctDNA test reporting** | | | | | |  |
| --- | --- | --- | --- | --- | --- | --- |
| **Statement** | **Strength of recommendation** | **Agree, essential**  **(no. of experts (%))** | **Agree, useful**  **(no. of experts (%))** | **Disagree**  **(no. of experts (%))** | **No opinion**  **(no. of experts (%))** | **Agreement (% (no. of experts agreeing/total no. of experts providing opinion))** |
| **Request for ctDNA testing** | | | | | |  |
| Patients, who are offered or opting for a liquid biopsy, should be informed regarding the possibility of unexpected and/or incidental findings and the (potential) consequences. | CR-E | 21 (48) | 20 (45) | 2 (5) | 1 (2) | 95 (41/43) |
| The request form should include an opt-out consent box to indicate whether the patient wants to be informed about unexpected and/or incidental findings. | CR-E | 18 (41) | 18 (41) | 6 (14) | 2 (5) | 86 (36/42) |
| Requests for ctDNA testing need to include the aim/purpose for ctDNA testing (e.g., identification of actionable targets in treatment-naive patient, identification of resistance mechanisms, monitoring). | CR-E | 27 (61) | 16 (36) | 0 (0) | 1 (2) | 100 (43/43) |
| **Availability of historical information prior to ctDNA testing** | | | | | |  |
| The following clinical information should be available (if known) prior to ctDNA testing, either in the request for ctDNA testing or otherwise: |  |  |  |  |  |  |
| - pathological diagnosis | CR-E | 31 (70) | 12 (27) | 0 (0) | 1 (2) | 100 (43/43) |
| - disease stage | CR-U | 15 (34) | 25 (57) | 2 (5) | 2 (5) | 95 (40/42) |
| - burden of disease (basic information on the number and localization of metastases) | CR-U | 8 (18) | 29 (66) | 5 (11) | 2 (5) | 88 (37/42) |
| - disease status (PD, SD, PR, CR) | CR-U | 17 (39) | 21 (48) | 3 (7) | 3 (7) | 93 (38/41) |
| - previous and current oncological treatment | CR-U | 15 (34) | 26 (59) | 2 (5) | 1 (2) | 95 (41/43) |
| - previously diagnosed malignancies | CR-U | 17 (39) | 23 (52) | 3 (7) | 1 (2) | 93 (40/43) |
| - diagnosed hereditary or confirmed tumor predisposition | CR-U | 12 (27) | 25 (57) | 4 (9) | 3 (7) | 90 (37/41) |
| The following molecular information should be available prior to ctDNA testing, either in the request for ctDNA testing or otherwise: |  |  |  |  |  |  |
| - known mutations from previous tissue profiling | CR-E | 26 (59) | 15 (34) | 3 (7) | 0 (0) | 93 (41/44) |
| - known mutations from previous liquid profiling | CR-E | 24 (55) | 16 (36) | 4 (9) | 0 (0) | 91 (40/44) |
| - previously identified CH-related mutations | CR-U | 18 (41) | 21 (48) | 3 (7) | 2 (5) | 93 (39/42) |
| The following clinical information should be stated in the final ctDNA report: |  |  |  |  |  |  |
| - pathological diagnosis | CR-E | 26 (59) | 14 (32) | 2 (5) | 2 (5) | 95 (40/42) |
| - disease stage | CR-U | 17 (39) | 18 (41) | 5 (11) | 4 (9) | 88 (35/40) |
| - burden of disease (basic information on the number and localization of metastases) | IR | 7 (16) | 22 (50) | 11 (25) | 4 (9) | 73 (29/40) |
| - disease status (PD, SD, PR, CR) | IR | 13 (30) | 17 (39) | 9 (20) | 5 (11) | 77 (30/39) |
| - previous and current oncological treatment | IR | 12 (27) | 19 (43) | 10 (23) | 3 (7) | 76 (31/41) |
| - previously diagnosed malignancies | IR | 8 (18) | 20 (45) | 10 (23) | 6 (14) | 74 (28/38) |
| - diagnosed hereditary or confirmed tumor predisposition | WR | 8 (18) | 18 (41) | 12 (27) | 6 (14) | 68 (26/38) |
| The following historical information should be stated in the final ctDNA report: |  |  |  |  |  |  |
| - known mutations from previous tissue profiling | CR-E | 21 (48) | 19 (43) | 3 (7) | 1 (2) | 93 (40/43) |
| - known mutations from previous liquid profiling | CR-E | 22 (50) | 18 (41) | 3 (7) | 1 (2) | 93 (40/43) |
| - previously identified CH-related mutations | CR-U | 11 (25) | 25 (57) | 5 (11) | 3 (7) | 88 (36/41) |
| **Pre-analytical variables and timeframe** | | | | | |  |
| Information of the following pre-analytical variables should be stated in the ctDNA report: |  |  |  |  |  |  |
| - type of collection tube(s) used | IR | 12 (27) | 18 (41) | 12 (27) | 2 (5) | 71 (30/42) |
| - date of blood sample collection | CR-E | 34 (77) | 10 (23) | 0 (0) | 0 (0) | 100 (44/44) |
| - date of storage of cell free plasma | WR | 11 (25) | 15 (34) | 15 (34) | 3 (7) | 63 (26/41) |
| - date of DNA/RNA isolation | WR | 5 (11) | 19 (43) | 16 (36) | 4 (9) | 60 (24/40) |
| - date of sample analysis | IR | 17 (39) | 15 (34) | 9 (20) | 3 (7) | 78 (32/41) |
| - date of report | CR-E | 34 (77) | 10 (23) | 0 (0) | 0 (0) | 100 (44/44) |
| **Assay specifications and performance assessment (methods)** | | | | | |  |
| The following technical assay specifications should be listed in the test report: |  |  |  |  |  |  |
| - specific test used | CR-E | 43 (98) | 1 (2) | 0 (0) | 0 (0) | 100 (44/44) |
| - scope of test | CR-E | 32 (73) | 10 (23) | 2 (5) | 0 (0) | 95 (42/44) |
| - method used for cfDNA isolation | CR-U | 14 (32) | 20 (45) | 8 (18) | 2 (5) | 81 (34/42) |
| - limit of detection (LOD) | CR-E | 36 (82) | 7 (16) | 1 (2) | 0 (0) | 98 (43/44) |
| - limit of quantification (LOQ) | CR-E | 18 (41) | 17 (39) | 6 (14) | 3 (7) | 85 (35/41) |
| - limit of blank (LOB) | WR | 15 (34) | 21 (48) | 6 (14) | 2 (5) | 86 (36/42) |
| - analytical sensitivity | CR-E | 25 (57) | 14 (32) | 5 (11) | 0 (0) | 89 (39/44) |
| - analytical specificity | CR-E | 23 (52) | 15 (34) | 6 (14) | 0 (0) | 86 (38/44) |
| - inter-assay variability | NR | 5 (11) | 17 (39) | 18 (41) | 4 (9) | 55 (22/40) |
| - intra-assay variability | NR | 4 (9) | 18 (41) | 18 (41) | 4 (9) | 55 (22/40) |
| - noise suppression method | NR | 2 (5) | 12 (27) | 21 (48) | 9 (20) | 40 (14/35) |
| - number and nature of reference materials and contrived samples that were used for assay validation | NR | 1 (2) | 12 (27) | 25 (57) | 6 (14) | 34 (13/38) |
| **Test results** | | | | | |  |
| The following quality metrics should be listed in the test report: |  |  |  |  |  |  |
| - macroscopic abnormalities of the blood sample (e.g., hemolysis) | CR-U | 15 (34) | 21 (48) | 5 (11) | 3 (7) | 88 (36/41) |
| - cfDNA concentration of the eluate | WR | 6 (14) | 21 (48) | 15 (34) | 2 (5) | 64 (27/42) |
| - cfDNA quantity (ng/mL) | IR | 11 (25) | 22 (50) | 10 (23) | 1 (2) | 77 (33/43) |
| - cfDNA integrity (if assessed; BioAnalyzer, Tapestation) | IR | 6 (14) | 27 (61) | 9 (20) | 2 (5) | 79 (33/42) |
| - library conversion rate of input molecules | NR | 4 (9) | 11 (25) | 20 (45) | 9 (20) | 43 (15/35) |
| - uniformity of coverage | WR | 10 (23) | 15 (34) | 14 (32) | 5 (11) | 64 (25/39) |
| - % of target region covered with the minimum required depth | CR-E | 20 (45) | 17 (39) | 6 (14) | 1 (2) | 86 (37/43) |
| If any of the above listed QC metrics do not meet the assay’s requirements, this should be clearly stated on the report. | CR-E | 35 (80) | 7 (16) | 2 (5) | 0 (0) | 95 (42/44) |
| **Reporting of variants & variants with low allelic frequency** | | | | | |  |
| For each variant the following parameter(s) should be reported: |  |  |  |  |  |  |
| - variant allele frequency (%) | CR-E | 38 (86) | 4 (9) | 1 (2) | 1 (2) | 98 (42/43) |
| - no. of mutated molecules | CR-E | 18 (41) | 14 (32) | 6 (14) | 6 (14) | 84 (32/38) |
| - sequencing depth | CR-E | 19 (43) | 19 (43) | 4 (9) | 2 (5) | 90 (38/42) |
| - base specific signal-to-noise ratio | NR | 3 (7) | 15 (34) | 17 (39) | 9 (20) | 51 (18/35) |
| - confidence level | CR-U | 13 (30) | 17 (39) | 7 (16) | 7 (16) | 81 (30/37) |
| Detected variants with an allelic frequency below or equal to the limit of blank (LOB), as validated by the diagnostic laboratory for the used test, should not be stated in the report. | CR-E | 27 (61) | 9 (20) | 5 (11) | 3 (7) | 88 (36/41) |
| Clinically relevant variants with variant allelic frequencies (VAFs) between the LOB and limit of detection (LOD) of the used test, if all quality parameters of the test have been met, should be reported. | IR | 18 (41) | 16 (36) | 10 (23) | 0 (0) | 77 (34/44) |
| A reported variant with allelic frequency between LOB and LOD should be labeled ‘equivocal variant’. There should be a disclaimer in the report stating the uncertainty of its presence. | IR | 16 (36) | 17 (39) | 8 (18) | 3 (7) | 80 (33/41) |
| Repeated or orthogonal testing methods should be performed to confirm the presence of equivocal variants. | CR-E | 17 (39) | 16 (36) | 4 (9) | 7 (16) | 89 (33/37) |
| Tissue and/or liquid re-biopsy should always be advised, if equivocal variants are reported. | CR-U | 15 (34) | 21 (48) | 3 (7) | 5 (11) | 92 (36/39) |
| If tumor fraction estimation is included in the test, mutation clonality versus subclonality should be approximated based on purity-normalized VAFs to account for variable tumor fractions. | CR-U | 5 (11) | 19 (43) | 3 (7) | 17 (39) | 89 (24/27) |
| Subclonal variants should be indicated as such. | IR | 6 (14) | 18 (41) | 9 (20) | 11 (25) | 73 (24/33) |
| If tumor fraction estimation is not included in the test, mutation clonality versus subclonality should not be approximated. | CR-E | 14 (32) | 11 (25) | 4 (9) | 15 (34) | 86 (25/29) |
| Variants in cancer susceptibility genes with VAFs indicating germline origin should be highlighted as such. | CR-E | 27 (61) | 12 (27) | 4 (9) | 1 (2) | 91 (39/43) |
| If a (potential) germline variant is reported, possible follow-up germline testing in the appropriate clinical context should be recommended. | CR-E | 30 (68) | 11 (25) | 3 (7) | 0 (0) | 93 (41/44) |
| **Distinguishing CH-related variants from tumor-derived variants** | | | | | |  |
| Additional analysis of PBMC-derived DNA should always be included to reliable distinguish between tumor-derived and clonal hemotapoiesis (CH)-related variants. | WR | 9 (20) | 15 (34) | 13 (30) | 7 (16) | 65 (24/37) |
| If a variant is suspected to originate from non-tumor sources and no PMBC are available, the variant should be flagged as a potential CH-related variant. | CR-U | 17 (39) | 22 (50) | 1 (2) | 4 (9) | 98 (39/40) |
| **SCNA and fusions** | | | | | |  |
| If the ctDNA assay enables somatic copy number alteration (SCNA) and fusion calling, the report should clearly state that the respective LOD are lower and their detection requires a high tumor fraction. |  | 17 (39) | 22 (50) | 2 (5) | 3 (7) | 95 (39/41) |
| For each SCNA the following parameters should be reported: |  |  |  |  |  |  |
| - estimated copy number | CR-E | 20 (45) | 17 (39) | 2 (5) | 5 (11) | 95 (37/39) |
| - estimated size of the amplified/deleted segment | CR-U | 8 (18) | 21 (48) | 6 (14) | 9 (20) | 83 (29/35) |
| - potentially co-amplified genes | CR-U | 7 (16) | 23 (52) | 6 (14) | 8 (18) | 83 (30/36) |
| - confidence level for each reported variant | CR-U | 13 (30) | 21 (48) | 2 (5) | 8 (18) | 94 (34/36) |
| **Reporting of negative results** | | | | | |  |
| If tumor fraction estimation is not included in the test, negative test results should be reported as 'ctDNA not detected'. Use of terms as 'wildtype', 'negative', or 'absence of mutation(s)' should be avoided. | CR-E | 35 (80) | 5 (11) | 3 (7) | 1 (2) | 93 (40/43) |
| If specific mutations were requested (i.e., KRAS ESR1, etc.) test results should be reported as 'requested mutation is not detected'. | CR-E | 33 (75) | 9 (20) | 2 (5) | 0 (0) | 95 (42/44) |
| Each report should include a disclaimer that the presence of mutations below the LOD cannot be excluded. | CR-E | 29 (66) | 12 (27) | 3 (7) | 0 (0) | 93 (41/44) |
| **Unexpected findings** | | | | | |  |
| Unexpected findings (i.e., somatic variant unfitting for pathological diagnosis/indicative of solid or hemaetological malignancy other than pathological diagnosis) should be accompanied by a disclaimer, including an explanation why the findings were unexpected. | CR-E | 22 (50) | 17 (39) | 2 (5) | 3 (7) | 95 (39/41) |
| Unexpected findings should standardly be referred to a Molecular Tumor Board for discussion. | CR-E | 23 (52) | 15 (34) | 4 (9) | 2 (5) | 90 (38/42) |
| CR-E, expert opinion consensus recommendation – essential element; CR-U, expert opinion consensus recommendation – useful element; IR, intermediate recommendation; WR, weak recommendation; NR, no recommendation; ctDNA, circulating tumor DNA; VUS, variants of unknown significance; LP/P, likely pathogenic/pathogenic; LOD, limit of detection; CHIP, clonal hematopoiesis of indeterminate potential; SCNA, somatic copy number alteration. | | | | | |  |

| **Overview of answers to multiple choice questions of the questionnaire** | |
| --- | --- |
| **Statement** | **No. of experts (%)** |
| **Reporting of variants + variants with low allelic frequency** |  |
| Please read all below statements regarding reporting of variants and choose one of the options. |  |
| Only pathogenic and likely pathogenic variants should be included in the report. | 20 (45) |
| Only pathogenic and likely pathogenic variants should be included in the main report. VUS should be listed as appendix. | 8 (18) |
| Only pathogenic and likely pathogenic variants should be included in the main report. Benign variants, likely benign variants and variants of unknown significance (VUS) should be listed as appendix. | 8 (18) |
| Only pathogenic variants, likely pathogenic and VUS should be included in the report. | 8 (18) |
| All variants, including benign and likely benign variants, should be included in the report. | 0 (0) |
| **Treatment recommendations** | |
| Please read all below statements regarding reporting of variants and choose one of the options. |  |
| Variants should only be matched with potential therapies in the report if evidence-based treatment recommendations (tier 1, ESCAT 1 or 2) are available. | 21 (48) |
| Treatment recommendations should only be listed for unequivocally actionable alterations. | 7 (16) |
| Treatment recommendations should never be included in the report. | 7 (16) |
| Clinical annotations should only be done by an MTB. | 9 (20) |
