# Supplementary material for: Reporting of molecular test results from cell-free DNA analyses: expert consensus recommendations from the 2023 European Liquid Biopsy Society ctDNA Workshop
Source: eBioMedicine. 2025 Mar 22;114:105636. doi: 10.1016/j.ebiom.2025.105636 (PMC11979934; doi:10.1016/j.ebiom.2025.105636)
Supplement: Supplementary File S3 [file mmc3.docx]

**Mock case #1**

**Case description**

A male patient of 65 years is diagnosed non-small cell lung cancer, cT2N1M1c, stage IV with brain metastases. He is a former smoker (5 pack years). There is no oncological medical history other than the current diagnosis. He has not yet received systemic treatment. No known hereditary or germline disease. At pathological diagnosis, there was insufficient tissue for predictive biomarker testing. The patient preferred liquid biopsy testing over tissue re-biopsy.

**Main results from diagnostic laboratory**

The results of cfDNA next-generation sequencing testing showed an *EGFR* p.(Leu858Arg) mutation with a variant allele frequency (VAF) of 0.14% (below the LOD). No variants are detected with VAF above the LOD of the test. All quality control metrics were met.

**Key points for reporting**

- The report should mention the LOB and LOD of the used test (Recommendation 6, Table 2);
- Variants with VAF between LOB and LOD (*EGFR* mutation in this case) should be labelled as ‘equivocal’ variant and there should be a disclaimer regarding the uncertainty of the presence of this mutation (Recommendation 10, Table 2);
- Orthogonal testing, tissue biopsy or liquid re-biopsy should be recommended to confirm the presence of the equivocal *EGFR* mutation (Recommendation 11, Table 2);
- Each report needs should state that the presence of mutations below the LOD cannot be excluded (Recommendation 21, Table 2).
- Variants with VAF below LOB should not reported.
- All tested variants in other regions within *EGFR* and in predictive markers other than *EGFR* should be reported as ‘not detected’. The use of terms as ‘wildtype’, ‘negative’ or ‘absence of mutation(s)’ should be avoided (Recommendation 19, Table 2).
- The *EGFR* p.(Leu858Arg) mutation is not suspected to be of hematopoietic origin (Table 3).

**Example report**

An example report, taking into account the recommendations, is provided on the following pages.

**Institute XXXXX**  Address: XXXXX

**Department of Pathology**

Prof. dr. XXXXX

Mol. Biol. Report L24-123456

Page 1 of 2

Concerning patient

XXXXX XXXXX

Date of birth: XX/XX/XXXX, Female

Patient ID nr.: PID-123456

Prof. dr. XXXXX SSN: XXXXXXXX

Laboratory for Molecular Pathology Department: XXXXX

A12345

Department

Institute City

Copy to:

Prof. dr. XXXXX

Requestor: Receipt of material : XX-XX-XXXX

Dr. XXXXX Date of report authorization : XX-XX-XXXX

Department of Oncology

Hospital XXXXX Medical admin. Tel. nr. : XXX-XXXXX

_______________________________________________________________________________________________________

**Copy Authorized report**

**Clinical information**

Patient with newly diagnosed non-small cell lung cancer, cT2N1M1c, stage IV with brain metastases. Former smoker (5 pack years). No oncological medical history other than current diagnosis. No systemic treatment received yet. No known hereditary or germline disease. At pathological diagnosis, there was insufficient tissue for predictive biomarker testing. Liquid biopsy was preferred by patient over tissue re-biopsy.

Test requested: ctDNA NGS.

□ Patient does not want to be informed about unexpected/incidental findings (cross if applicable).

**Macroscopy**

XX-XX-XXXX: Received material: 10mL blood, Hospital XXXXX, PID-123456

**Summary results:**

| ***EGFR* mutation:** | L858R (equivocal, <LoD95) |
| --- | --- |
| **Other actionable mutation:** | not detected |
| **Clinically relevant copy number alteration:** | not detected |

**Mutations:**

| **Gen** | **Variante**^1^ | **VAF**^2^ | **Sequence Depth**^3^ | **Classification**^4^ | **Comments** |
| --- | --- | --- | --- | --- | --- |
| *EGFR* | NM_005228.5:  c.2573T>G p.(Leu858Arg) | 0.14% | 4/2867 | **pathogenic**  [GOF] | equivocal, VAF <LOD95* |

**^1^**According to HGVS nomenclature; **^2^**VAF, Variant allele frequency; **^3^** Sequencing depth indicates how often the respecitive position in the genome was sequenced - the number of mutated or the number of sequenced fragments is indicated; **^4^**Variants are classified according to the ACMG/AMP standards. LOF, loss-of-function, GOF, gain-of-function. *LOD95 is the lowest VAF at which mutations are detected with 95% probability. CH, clonal hematopoesis

***For detailed clinical annotation of the detected variants please refer to a Molecular Tumor Board!***

**Interpretation:**

A therapy-relevant mutation in the *EGFR* gene with a VAF of 0.14% was detected, which is below the specified detection limit, at which mutations are detected with 95% probability. Re-testing in 6-8 weeks is recommended to confirm its presence.

Note: The presence of variants below the detection limit or in genes not examined cannot be excluded. In addition, indels are only called in selected genes (see appendix). This test enable the detection of both germline and somatic variants. Germline alterations that are currently interpreted as functional or disease-associated polymorphisms or as “sequence variants of uncertain clinical significance”, “likely neutral” or “neutral” are generally not listed in the findings.

**Method:**

Extraction of plasma from whole blood using the double-spin protocol and subsequent isolation of cell-free DNA from blood plasma using *XXX* cfDNA Isolation Kit.

Enrichment of a total of >500 genes ( X Mb) using the XXXX ctDNA enrichment technology (Vendor) and sequencing on the Illumina platform. Analysis is performed using the ctDNA Analysis Software XXXX, followed by filtering of sequence alterations based on their frequency in the general population (<1%), variant allele frequency (VAF, at least 0.1% of sequenced fragments must carry the sequence alteration), and quality parameters (PASS). Intron variants outside of splice sites as well as benign variants are not reported. As part of the technical evaluation, using 20ng of cfDNA, a 95% sensitivity was demonstrated for detecting sequence variants (SNVs/Indels) with a VAF of 0.25% at a detection limit (LOD) of 0.1%. Fusions can be detected at an LOD of 0.5% with a sensitivity of 73%. Lower input amounts may negatively affect sensitivity. Additionally, with sufficient tumor content (>3%), MSI, TMB, rearrangements, and copy number alterations can also be detected. Interpretation of variants according to ACMG guidelines. Unless otherwise stated, only pathogenic and likely pathogenic variants (class 4 and 5) are reported in the context of the clinical question. Assessed genes for this request: SNVs/Indels in *BRAF* (NM_004333.6), *EGFR* (NM_005228.5), *ERBB2* (NM_004448.4), *KRAS* (NM_004985.5), *PIK3CA* (NM_006218.4), *MET* (NM_000245.4), *TP53* (NM_000546.6), *STK11* (NM_000455.5), *KEAP1* (NM_203500.2), and gene-fusion rearrangements involving *ALK*, *ROS1*, *RET*, *NTRK1*, *NTRK2*, *NTRK3*, *FGFR1*, *FGFR2*, *FGFR3* and *NRG1*.

Limitations:

The presence of mutations with VAF below the detection limit of 0.5% cannot be excluded.

Literature references:

ACMG Standards (PMID: 25741868) or AMP Standards (PMID: 27993330).

**APPENDIX:**

Present copy number profile

List VUS

Present list of genes that have been analyzed

**OPTIONAL DESCRIPTION OF VARIANTS**

**NM_005228.5 (*EGFR*): c.2573T>G p.(Leu858Arg) [L858R]; VAF 0.14%**

This variant is a common *EGFR* mutation in the kinase domain. *EGFR* mutations occur in approximately 10% of European patients with metastatic non-small cell lung cancer (NSCLC) (higher prevalence in Asian patients). For patients with metastatic NSCLC harboring a common *EGFR* mutation, multiple EGFR tyrosine kinase inhibitors have been approved as first-line treatment option by the European Medicines Agency (EMA).
