# Supplementary material for: Reporting of molecular test results from cell-free DNA analyses: expert consensus recommendations from the 2023 European Liquid Biopsy Society ctDNA Workshop
Source: eBioMedicine. 2025 Mar 22;114:105636. doi: 10.1016/j.ebiom.2025.105636 (PMC11979934; doi:10.1016/j.ebiom.2025.105636)
Supplement: Supplementary File S4 [file mmc4.docx]

**Mock case #2**

**Case description**

A female patient of 56 years is diagnosed with locally advance hormone receptor positive (HR+), HER2-negative breast cancer. After endocrine and CDK4/6 treatments, she presented progressive disease. The attending physician has requested *ESR1* testing from plasma to evaluate a potential therapy adjustment to a Selective Estrogen Receptor Degrader (SERD).

**Main results from diagnostic laboratory**

NSG testing including 77 clinically relevant genes and shallow whole genome sequencing (sWGS) to infer somatic copy number alterations (SCNA) and tumor fraction.

A subclonal *ESR1* mutation (< LoD95) was identified at an estimated tumor fraction of 17%. Moreover, *PIK3CA* hotspot mutation was detected with a VAF in accordance with the estimated tumor content. In addition, a pathogenic *BRCA1* mutation that considered alongside the tumor content suggests the presence in the germline, was identified, which should be followed in with germline testing.

**Key points for reporting**

- The report should mention the LOB and LOD of the used test (Recommendation 6, Table 2);
- Variants with VAF, in this case the ESR1 mutation, between LOB and LOD should be labelled as ‘equivocal’ variant and there should be a disclaimer regarding the uncertainty of the presence of this mutation (Recommendation 10, Table 2).
- Orthogonal testing, tissue biopsy or liquid re-biopsy should be recommended to confirm the presence of the equivocal *ESR1* mutation (Recommendation 14, Table 2);
- Variants in cancer susceptibility genes with VAF indicating germline origin should be highlighted as such (Recommendation 15, Table 2).
- If a putative germline variant is reported, genetic counselling and/or germline testing should be recommended (Recommendation 11, Table 2).
- Without PBMC-testing to correct for CH-related variants, variants suspected to originate from non-tumor sources should be flagged as a ‘potential CH-related variant (Recommendation 16, Table 2).
- Each report needs should state that the presence of mutations below the LOD cannot be excluded (Recommendation 21, Table 2).
- Variants with VAF below LOB should not reported.
- The *ESR1* p.(Glu380Gln) mutation is not suspected to be of hematopoietic origin, since it is a known mutation that is acquired as a consequence of the selective pressure of endocrine treatment (Table 3). In this case, the mutation is likely about to emerge and a subclonal presence is not unexpected.

**Example report**

An example report, taking into account the recommendations, is provided on the following two pages.

**Institute XXXXX**  Address: XXXXX

**Department of Pathology**

Prof. dr. XXXXX

Mol. Biol. Report L24-123456

Page 1 of 2

Patient NAME

Date of birth: DD/MM/JJJJ, Female

Patient ID nr.: PID-123456

Prof. dr. XXXXX SSN: XXXXXXXX

Laboratory for Molecular Pathology Department: XXXXX

A12345

Department

Institute City

Copy to:

Prof. dr. XXXXX

Requestor: Receipt of material : XX-XX-XXXX

Dr. XXXXX Date of report authorization : XX-XX-XXXX

Department of Oncology

Hospital XXXXX Medical admin. Tel. nr. : XXX-XXXXX

_______________________________________________________________________________________________________

**Copy Authorized report**

**Clinical information**

Female, 56 years, HR+ HER2-negative breast cancer, endocrine resistance.

Potential therapy adjustment to a Selective Estrogen Receptor Degrader (SERD).

Screening for acquired resistance mutation in ESR1, liquid biopsy preferred

Test requested: ctDNA NGS.

□ Patient does not want to be informed about unexpected/incidental findings (cross if applicable).

**Macroscopy**

XX-XX-XXXX: Received material: 10mL blood, Hospital XXXXX, PID-123456

**Summary results:**

| **Estimated tumor fraction (according to ichorCNA):** | 17% |
| --- | --- |
| ***ESR1* mutation:** | E380Q (equivocal, <LoD95) |
| **Other actionable mutation:** | *BRCA1* C61G (suspected germline variant) |
| **Clinically relevant copy number alteration:** | not detected |

**Mutations:**

| **Gen** | **Variante**^1^ | **VAF**^2^ | **Sequence Depth**^3^ | **Classification**^4^ | **Comments** |
| --- | --- | --- | --- | --- | --- |
| *ESR1* | NM_000125.4:  c.1138G>C, p.(Glu380Gln) | 0.3% | 11/3815 | **pathogenic**  [GOF] | subclonal, VAF <LOD95* |
| *PIK3CA* | NM_006218.4:  c.1624G>A, p.(Glu542Lys) | 12.3% | 239/1951 | **pathogenic**  [GOF] | none |
| *BRCA1* | NM_007294.4:  c.181T>G, (p.Cys61Gly) | 62.7% | 1383/2207 | **pathogenic**  [LOF] | most likely germline origin |
| *TP53* | NM_000546.6:  c.841G>A, p.(Asp281Asn) | 0.5% | 14/2810 | **pathogenic**  [LOF] | subclonal or CH-related |

**^1^**According to HGVS nomenclature; **^2^**VAF, Variant allele frequency; **^3^** Sequencing depth indicates how often the respecitive position in the genome was sequenced - the number of mutated or the number of sequenced fragments is indicated; **^4^**Variants are classified according to the ACMG/AMP standards. LOF, loss-of-function, GOF, gain-of-function. *LOD95 is the lowest VAF at which mutations are detected with 95% probability. CH, clonal hematopoesis

***For detailed clinical annotation of the detected variants please refer to a Molecular Tumor Board!***

**Interpretation:**

At an estimated tumor fraction of cell-free DNA from plasma of 17%, a therapy-relevant mutation in the *ESR1* gene with a VAF of 0.3% was detected, which is below the specified detection limit, at which mutations are detected with 95% probability. Re-testing in 6-8 weeks is recommended to confirm the presence.

In addition, further pathogenic mutations were detected in the *PIK3CA* and *BRCA1* genes. Considering the tumor fraction, the *BRCA1* mutation is likely of germline origin, which is why genetic counselling and germline testing is highly recommended.

Moreover, a low level TP53 mutation was identified with a VAF of 0.5%. Low-frequency variants can, however, also represent clonal hematopoiesis and may not originate from the tumor.

Although a variety of copy number changes were detected by with sWGS, no clinically relevant changes were identified.

Note: The presence of variants below the detection limit or in genes not examined cannot be excluded. In addition, indels are only called in selected genes (see appendix). This test enable the detection of both germline and somatic variants. Germline alterations that are currently interpreted as functional or disease-associated polymorphisms or as “sequence variants of uncertain clinical significance”, “likely neutral” or “neutral” are generally not listed in the findings.

**Method:**

Extraction of plasma from whole blood using the double-spin protocol and subsequent isolation of cell-free DNA from blood plasma using *XXX* cfDNA Isolation Kit.

Enrichment and sequencing on the Illumina platform of a total of **kb of 77 genes including those in the guidelines of the U.S. National Comprehensive Cancer Network (NCCN) as well as biomarkers relevant for clinical research using the ctDNA NGS Assay Kit (*Company*). Data analysis is carried out via a bioinformatics pipeline from *** version * and subsequent filtering of sequence changes with regard to population allele frequency (MAF<1%) and variant allele frequency (VAF, at least 0.1%. Technical evaluation revealed a LoD95 at aVAF of 0.5%. However, the detection of copy number changes and rearrangements is impaired at low tumor fractions. Variants with less than 10 mutated fragments are only reported in cases of clinical relevance. Intron variants outside splice sites and benign variants are not reported.

Genomewide copy number calling is based on read count analysis from shallow whole genome sequencing (REF), Genome Med 2013). Identification of focal alterations from segmented copy number data. Criteria for a focal events: segment size < 20Mb; log2 ratios >0.2 or <-0.2; < 100 genes in the segment; difference in log2 ratio to neighboring segment >0.2 if known tumor driver gene affected or >0.58 for unknown tumor driver genes (REF). Estimation of tumor content using the ichorCNA algorithm (REF). Tumor fractions of <3% are not informative.

Limitations:

The presence of mutations with VAF below the detection limit of 0.5% cannot be excluded.

Literature references:

ACMG Standards (PMID: 25741868) or AMP Standards (PMID: 27993330)

**APPENDIX:**

Present copy number profile

List VUS

Present list of genes that have been analyzed

**OPTIONAL DESCRIPTION OF VARIANTS**

**NM_000125.4 (ESR1): c.1138G>C, p.(Glu380Gln) [E380Q]; VAF 0.3%***

The alteration represents one of the most common *ESR1* mutations in the ligand-binding domain. *ESR1* mutations occur in up to 40% of patients with ER+, HER2-, locally advanced, or metastatic breast cancer and are considered a cause of resistance to standard endocrine therapy. Patients with confirmed activating *ESR1* mutations in plasma can be treated with the selective estrogen receptor degrader (SERD) Elacestrant, as its administration leads to significantly prolonged progression-free survival compared to standard treatment (PMID: 35584336).

**NM_006218.4(*PIK3CA*): c.1624G>A, p.(Glu542Lys) [E542K]; VAF 12.3%**

This variant is a known gain-of-function (GOF) hotspot mutation in the helical domain of the protein and is among the most common *PIK3CA* mutations. Approximately 40% of all hormone receptor-positive breast carcinomas exhibit activating mutations in the *PIK3CA* gene, which can be specifically inhibited by phosphoinositide 3-kinase (PI3K) inhibitors. The PI3K inhibitor Alpelisib, in combination with the antiestrogen Fulvestrant, has nearly doubled the progression-free survival of patients with hormone receptor-positive, HER2-negative breast carcinoma with PIK3CA mutations in a Phase III study.

**NM_007294.4 (*BRCA1*): c.181T>G, p.(Cys61Gly), [C61G]; VAF 48.8%**

The alteration is a missense mutation that disrupts the heterodimerization of the protein with BARD1. The variant is known in international databases and is described as a founder mutation in the Polish, German, Czech, and Austrian populations, as it has been identified in numerous hereditary breast and/or ovarian cancer families (ClinVar Variation ID: 17661). This mutation is therefore classified as pathogenic**.**

**Based on the estimated tumor fraction, it is presumed to be present in the germline.** The identification of a pathogenic germline mutation in the BRCA1 gene significantly increases the lifetime risk of developing breast and/or ovarian cancer. Additionally, there is an elevated risk for other cancers (e.g., pancreatic and prostate cancers). For this reason, an intensive genetic counselling and germline testing is strongly recommended.

The PARP inhibitors Olaparib and Talazoparib are approved for the treatment of patients with HER2-negative metastatic breast cancer who have germline mutations in BRCA1/2. BRCA1 encodes a tumor suppressor involved in the DNA damage response and is mutated in various types of cancer.

**NM_000546.6(*TP53*):c.841G>A, p.(Asp281Asn) [D281N]; VAF 0.5%**

This mutation is a missense variant in the DNA-binding domain that, according to in vivo and in vitro studies leads to a loss of protein function (LOF, loss-of-function) (PMID: 27328919, 12826609, 29979965, 30224644). *TP53* is the most frequently mutated gene in cancer; however, there are currently no effective therapeutic approaches targeting it.
