# Supplementary material for: Reporting of molecular test results from cell-free DNA analyses: expert consensus recommendations from the 2023 European Liquid Biopsy Society ctDNA Workshop
Source: eBioMedicine. 2025 Mar 22;114:105636. doi: 10.1016/j.ebiom.2025.105636 (PMC11979934; doi:10.1016/j.ebiom.2025.105636)
Supplement: Supplementary File S5 [file mmc5.docx]

**Mock case #3**

**Case description**

A male patient of 73 diagnosed with stage IV CRC and liver metastases. The primary tumor did not carry *RAS* mutations, which is why the patient was eligible for anti-EGFR treatment. After an initial response, the patient developed resistance based on a *KRAS* G12D mutation and treatment was discontinued 6 months ago. The attending physician has requested *KRAS* testing to see if the resistant clones have lost their selective advantage and diminished in the absence of treatment pressure in order to re-administration (re-challenge) of anti-EGFR treatment.

**Main results from diagnostic laboratory**

NSG testing including 77 clinically relevant genes and shallow whole genome sequencing (sWGS) to infer somatic copy number alterations (SCNA) and tumor fraction. In accordance with the estimated tumor fraction of 16%, two pathogenic mutation in *APC* and *TP53* were detected. Moreover, a focal amplification of the *ERBB2* gene (HER2) was identified. Given the high tumor fraction, it can be assumed that the mutated *KRAS* clone has disappeared.

**Key points for reporting**

- The report should mention the LOB and LOD of the used test (Recommendation 6, Table 2);
- Each report needs should state that the presence of mutations below the LOD cannot be excluded (Recommendation 21, Table 2).
- Variants with VAF below LOB should not reported.
- The *TP53* p.(R175H) mutation is not suspected to be of hematopoietic origin, since its VAF is consistent with the estimated tumor content and was not detected in the previous liquid biopsy (Table 3).
- If specific mutations were requested (in this case *KRAS*), test results should be reported as ‘requested mutation is not detected’ (Recommendation 20, Table 2).
- For SCNA (in this case *ERBB2* amplification), the estimated copy number or log2 ratio, confidence level, potentially co-amplified genes and estimated size of the amplified/deleted segment should be reported (Recommendation 18, Table 2).

**Example report**

An example report, taking into account the recommendations, is provided on the following pages.

**Institute XXXXX**  Address: XXXXX

**Department of Pathology**

Prof. dr. XXXXX

Mol. Biol. Report L24-123456

Page 1 of 2

Patient NAME

Date of birth: DD/MM/JJJJ, Female

Patient ID nr.: PID-123456

Prof. dr. XXXXX SSN: XXXXXXXX

Laboratory for Molecular Pathology Department: XXXXX

A12345

Department

Institute City

Copy to:

Prof. dr. XXXXX

Requestor: Receipt of material : XX-XX-XXXX

Dr. XXXXX Date of report authorization : XX-XX-XXXX

Department of Oncology

Hospital XXXXX Medical admin. Tel. nr. : XXX-XXXXX

_______________________________________________________________________________________________________

**Copy Authorized report**

**Clinical information**

Male, 73 years, stage IV CRC and liver metastases

Progression on anti-EGFR treatment due to emergence *KRAS* G12D mutation, treatment holiday for 6 month.

Neo RAS WT testing for potential on anti-EGFR rechallenge is requested

Test requested: ctDNA NGS.

□ Patient does not want to be informed about unexpected/incidental findings (cross if applicable).

**Macroscopy**

XX-XX-XXXX: Received material: 10mL blood, Hospital XXXXX, PID-123456

**Summary results:**

| **Estimated tumor fraction (according to ichorCNA):** | 16% |
| --- | --- |
| ***KRAS* mutation:** | not detected |
| **Other actionable mutation:** | not detected |
| **Clinically relevant copy number alteration:** | *ERBB2* amplification |

**Mutations:**

| **Gen** | **Variante**^1^ | **VAF**^2^ | **Sequence Depth**^3^ | **Classification**^4^ | **Comments** |
| --- | --- | --- | --- | --- | --- |
| *APC* | NM_000038.5:  c.4561G>T, p.(Glu1521*) | 37.2% | 1095/2945 | **pathogenic**  [GOF] | none |
| *TP53* | NM_006218.4:  c.524G>A (p.Arg175His) | 29.5% | 634/2151 | **pathogenic**  [GOF] | none |

**^1^**According to HGVS nomenclature; **^2^**VAF, Variant allele frequency; **^3^** Sequencing depth indicates how often the respecitive position in the genome was sequenced - the number of mutated or the number of sequenced fragments is indicated; **^4^**Variants are classified according to the ACMG/AMP standards. LOF, loss-of-function, GOF, gain-of-function. *LOD95 is the lowest VAF at which mutations are detected with 95% probability.

**Focal copy number alterations:**

| **Chrom^1^** | **Start^1^** | **End^1^** | **Size [Mb]^2^** | **Log2ratio^3^** | **Type^4^** | **No. of genes^5^** | **Clinically relevant genes** |
| --- | --- | --- | --- | --- | --- | --- | --- |
| Chr17 | 36950811 | 38586699 | 1.6 | 1.2 | Amp | 53 | *ERBB2* |

^1^Position of the amplified/deleted genomic segment [hg19]; ^2^Size of the genomic segment in megabases (Mb); ^3^Log2-Ratios of the segment der Segmente; 4Amplification (Amp), Deletion (Del); ^5^ Number of gene in the segment.

***For detailed clinical annotation of the detected variants please refer to a Molecular Tumor Board!***

**Interpretation:**

At an estimated tumor fraction of cell-free DNA from plasma of 16% neither the previously identified G12D *KRAS* mutation nor any other RAS mutation was identified.

However, pathogenic mutations in the genes *APC* and *TP53* were detected consistent with the estimated tumor content. Using shallow whole-genome sequencing (sWGS), a focal amplification on chromosome 17q12 was identified, including the *ERBB2* gene.

Note: The presence of variants below the detection limit or in genes not examined cannot be excluded. In addition, indels are only called in selected genes (see appendix). This test enable the detection of both germline and somatic variants. Germline alterations that are currently interpreted as functional or disease-associated polymorphisms or as “sequence variants of uncertain clinical significance”, “likely neutral” or “neutral” are generally not listed in the findings.

**Method:**

Extraction of plasma from whole blood using the double-spin protocol and subsequent isolation of cell-free DNA from blood plasma using *XXX* cfDNA Isolation Kit.

Enrichment and sequencing on the Illumina platform of a total of **kb of 77 genes including those in the guidelines of the U.S. National Comprehensive Cancer Network (NCCN) as well as biomarkers relevant for clinical research using the ctDNA NGS Assay Kit (*Company*). Data analysis is carried out via a bioinformatics pipeline from *** version * and subsequent filtering of sequence changes with regard to population allele frequency (MAF<1%) and variant allele frequency (VAF, at least 0.1%. Technical evaluation revealed a LoD95 at aVAF of 0.5%. However, the detection of copy number changes and rearrangements is impaired at low tumor fractions. Variants with less than 10 mutated fragments are only reported in cases of clinical relevance. Intron variants outside splice sites and benign variants are not reported.

Genomewide copy number calling is based on read count analysis from shallow whole genome sequencing (REF), Genome Med 2013). Identification of focal alterations from segmented copy number data. Criteria for a focal events: segment size < 20Mb; log2 ratios >0.2 or <-0.2; < 100 genes in the segment; difference in log2 ratio to neighboring segment >0.2 if known tumor driver gene affected or >0.58 for unknown tumor driver genes (REF). Estimation of tumor content using the ichorCNA algorithm (REF). Tumor fractions of <3% are not informative.

Limitations:

The presence of mutations with VAF below the detection limit of 0.5% cannot be excluded.

Literature references:

ACMG Standards (PMID: 25741868) or AMP Standards (PMID: 27993330)

**APPENDIX:**

Present copy number profile

List VUS

Present list of genes that have been analysed

**OPTIONAL DESCRIPTION OF VARIANTS**

**NM_000038.6 (*APC*): c.4561G>T, p.(Glu1521*) [E1281X]; VAF 37.2%**

The variant in the is a nonsense mutation (LOF, loss-of-function) resulting in a premature stop codon. This either leads to an early termination of protein synthesis or to nonsense-mediated mRNA decay, where the defective mRNA is degraded prematurely. APC encodes a tumor suppressor involved in WNT signaling and is frequently mutated in colorectal cancer. *APC* is a tumor suppressor involved in WNT signaling and is very frequently inactivated in colorectal carcinomas. Currently, there are no effective therapeutic approaches for APC-mutated tumors.

**NM_000546.6(*TP53*):c.524G>A (p.Arg175His); [R175H]; VAF 29.5%**

This mutation is a well characterized missense variant in the DNA-binding domain that leads to a loss of protein function (LOF, loss-of-function) ((PMID: 15781620, 25584008, 31068365). *TP53* is the most frequently mutated gene in cancer; however, there are currently no effective therapeutic approaches targeting it.

**Amplification of Chr17q12 including *ERBB2* (HER2)**

An amplification of *ERBB2* leads to overexpression of the protein, resulting in increased activation of signaling pathways (PMID: 23204226, 12124352, 11571643, 10716706, 2885917). The therapeutics tucatinib and trastuzumab deruxtecan are approved for the treatment of patients with HER2-postitive solid tumors and NCCN-compendium listed for the treatment of patients with HER2-amplified colorectal cancer.
