# Supplementary material for: Reporting of molecular test results from cell-free DNA analyses: expert consensus recommendations from the 2023 European Liquid Biopsy Society ctDNA Workshop
Source: eBioMedicine. 2025 Mar 22;114:105636. doi: 10.1016/j.ebiom.2025.105636 (PMC11979934; doi:10.1016/j.ebiom.2025.105636)
Supplement: Supplementary File S6 [file mmc6.docx]

**Mock case #4**

**Case description**

A male patient of 73 diagnosed with stage IV CRC and liver metastases. The primary tumor did not carry *RAS* mutations, which is why the patient was eligible for anti-EGFR treatment. After an initial response, the patient developed resistance based on a *KRAS* G12D mutation and treatment was discontinued 6 months ago. The attending physician has requested *KRAS* testing to see if the resistant clones have lost their selective advantage and diminished in the absence of treatment pressure in order to re-administration (re-challenge) of anti-EGFR treatment.

**Main results from diagnostic laboratory**

NSG testing including 77 clinically relevant genes and shallow whole genome sequencing (sWGS) to infer somatic copy number alterations (SCNA) and tumor fraction. In accordance with the estimated tumor fraction of 16%, two pathogenic mutation in *APC* and *TP53* were detected. Moreover, a focal amplification of the *ERBB2* gene (HER2) was identified. Given the high tumor fraction, it can be assumed that the mutated *KRAS* clone has disappeared.

**Key points for reporting**

- The report should mention the LOB and LOD of the used test (Recommendation 6, Table 2);
- Without PBMC-testing to correct for CH-related variants, variants suspected to originate from non-tumor sources should be flagged as a ‘potential CH-related variant (Recommendation 16, Table 2).
- Each report needs should state that the presence of mutations below the LOD cannot be excluded (Recommendation 21, Table 2).
- Variants with VAF below LOB should not reported.
- The *JAK2* p.(E1521*) mutation and the *TET2* p.(K1243fs) mutation are likely of hematopoietic origin, due to their association with CHIP and their VAFs (Table 3).
- Clinically actionable results and evidence-based associations with response to specific drugs should be disclosed, but treatment recommendations should not be given (Recommendation 24, Table 2).

**Example report**

An example report, taking into account the recommendations, is provided on the following pages.

**Institute XXXXX**  Address: XXXXX

**Department of Pathology**

Prof. dr. XXXXX

Mol. Biol. Report L24-123456

Page 1 of 2

Patient NAME

Date of birth: DD/MM/JJJJ, Female

Patient ID nr.: PID-123456

Prof. dr. XXXXX SSN: XXXXXXXX

Laboratory for Molecular Pathology Department: XXXXX

A12345

Department

Institute City

Copy to:

Prof. dr. XXXXX

Requestor: Receipt of material : XX-XX-XXXX

Dr. XXXXX Date of report authorization : XX-XX-XXXX

Department of Oncology

Hospital XXXXX Medical admin. Tel. nr. : XXX-XXXXX

_______________________________________________________________________________________________________

**Copy Authorized report**

**Clinical information**

Female, 59 years, bile duct cancer, stage IV (cT4N1M1c)

Several lines of previous chemotherapies, currently progression

Identification of actionable targets

Test requested: ctDNA NGS.

□ Patient does not want to be informed about unexpected/incidental findings (cross if applicable).

**Macroscopy**

XX-XX-XXXX: Received material: 10mL blood, Hospital XXXXX, PID-123456

**Summary results:**

| **Estimated tumor fraction:** | 2% |
| --- | --- |
| **Tumor Mutational Burden (TMB):** | 1 Muts/Mb |
| **Microsatellite status:** | stable (MSS) |
| **Clinically relevant mutations:** | not detected |
| **Clinically relevant copy number alteration:** | not detected |
| **Fusions:** | *FGFR2*-*BICC1* fusion |

**Mutations:**

| **Gen** | **Variante**^1^ | **VAF**^2^ | **Sequence Depth**^3^ | **Classification**^4^ | **Comments** |
| --- | --- | --- | --- | --- | --- |
| *JAK2* | NM_000038.5:  c.4561G>T, p.(Glu1521*) | 47.2% | 1084/2298 | **pathogenic**  [LOF] | likely CH-related |
| *BAP1* | NM_004656.4:  c.2097del, p.(Arg700Glyfs*36) | 1.2% | 20/1685 | **pathogenic**  [LOF] | none |
| *TET2* | NM_001127208.3:  c.3727_3728del, p.Lys1243Thrfs*24 | 1.1% | 22/2065 | **pathogenic**  [LOF] | likely CH-related |

**^1^**According to HGVS nomenclature; **^2^**VAF, Variant allele frequency; **^3^** Sequencing depth indicates how often the respecitive position in the genome was sequenced - the number of mutated or the number of sequenced fragments is indicated; **^4^**Variants are classified according to the ACMG/AMP standards. LOF, loss-of-function, GOF, gain-of-function. *LOD95 is the lowest VAF at which mutations are detected with 95% probability.

**Fusions:**

| **Gen 1** | **Gen 2** | **Break-end 1** | **Break-end 2** | **Supporting Reads** | **Total Reads** | **VAF**^1^ | **Fusion** |
| --- | --- | --- | --- | --- | --- | --- | --- |
| *FGFR2* | *BICC1* | chrN: 123456789 | chrN: 123456789 | 32 | 4101 | 0.8% | *FGFR2-BICC1* |

**^1^**VAF, Variant allele frequency

***For detailed clinical annotation of the detected variants please refer to a Molecular Tumor Board!***

**Interpretation:**

At an estimated tumor fraction of cell-free DNA from plasma of 2% , a pathogenic mutations in the *BAP1* gene as well as a *FGFR2-BICC1* fusion with a VAF of 0.8% could be identified.

Moreover, pathogenic mutations in *JAK2* and *TET2* were detected, which are most likely of hematopoietic origin. The high VAF of the *JAK2* variant [V617F] of 47.2% suggests the presence of a myeloproliferative neoplasm (MPN), possibly as a consequence of chemotherapy. The *JAK2* V617F mutation is detectable in over 90% of patients with polycythemia vera (PV) as well as in 50% to 60% of patients with essential thrombocythemia and chronic idiopathic myelofibrosis. Case studies, including the present case, have reported the concurrent presence of the mutation and a Chr3p12-14 deletion, which, in addition to PV, has been linked to the development of solid tumors (Ayvaz et al., J Gastrointest Cancer, 2010; Rai et al., IJSR, 2024).

Note: The presence of variants below the detection limit or in genes not examined cannot be excluded. In addition, indels are only called in selected genes (see appendix). This test enable the detection of both germline and somatic variants. Germline alterations that are currently interpreted as functional or disease-associated polymorphisms or as “sequence variants of uncertain clinical significance”, “likely neutral” or “neutral” are generally not listed in the findings.

**Method:**

Extraction of plasma from whole blood using the double-spin protocol and subsequent isolation of cell-free DNA from blood plasma using *XXX* cfDNA Isolation Kit.

Enrichment of a total of >500 genes ( X Mb) using the XXXX ctDNA enrichment technology (Vendor) and sequencing on the Illumina platform. Analysis is performed using the ctDNA Analysis Software XXXX, followed by filtering of sequence alterations based on their frequency in the general population (<1%), variant allele frequency (VAF, at least 0.1% of sequenced fragments must carry the sequence alteration), and quality parameters (PASS). Intron variants outside of splice sites as well as benign variants are not reported. As part of the technical evaluation, using 20ng of cfDNA, a 95% sensitivity was demonstrated for detecting sequence variants (SNVs/Indels) with a VAF of 0.25% at a detection limit (LOD) of 0.1%. Fusions can be detected at an LOD of 0.5% with a sensitivity of 73%. Lower input amounts may negatively affect sensitivity. Additionally, with sufficient tumor content (>3%), MSI, TMB, rearrangements, and copy number alterations can also be detected.

Limitations:

The presence of mutations with VAF below the detection limit (0.1% for SNVs/Indels, 0.5% for fusions) cannot be excluded.

Literature references:

ACMG Standards (PMID: 25741868) or AMP Standards (PMID: 27993330).

**APPENDIX:**

Present copy number profile

List VUS

Present list of genes that have been analysed

**OPTIONAL DESCRIPTION OF VARIANTS**

**NM_004656.4(*BAP1*):c.2097del, p.(Arg700Glyfs*36)) [R700fs]; VAF 1.2%**

The variant is a frameshift mutation resulting in a premature stop codon. This leads either to an early termination of protein synthesis or to nonsense-mediated mRNA decay, where the defective mRNA is prematurely degraded. *BAP1* is a tumor suppressor and deubiquitinating enzyme.

**NM_004972.4(JAK2): c.1849G>T, p.(Val617Phe) [V617F]; VAF 47.2%**

The mutation an oncogenic missense variant in the tyrosine kinase domain of the protein, disrupting the autoinhibitory JH2 function (PMID: 12351625, 21533163). This mutation is the most common *JAK2* mutation and occurs in combination with *IDH1/2* mutations in myeloproliferative neoplasms. In vivo studies have shown that combined inhibition of *JAK2* and I*DH1/2* with ruxolitinib and enasidenib in this context results in a stronger reduction of tumor burden than either drug alone (PMID: 29355841). Moreover, it should be noted that a recent study demonstrated that up to 8% of healthy individuals carry *JAK2* mutations associated with CHIP (clonal hematopoiesis of indeterminate potential) (PMID: 32761230). While *JAK2* V617F mutations are predictive of response to JAK inhibitors in patients with myelofibrosis, there is currently no evidence that a JAK2 mutation is predictive of response in pancreatic cancer (Sochacki et al., Blood 2019). However, it is hypothesized that patients with elevated CRP levels, whose inflammatory response appears to be mediated by the JAK/STAT signaling pathway, could benefit from JAK inhibitors (Garrido-Laguna, Contemporary Oncology 2015).

**NM_001127208.3(*TET2*): c.3727_3728del, p.Lys1243Thrfs*24 [K1243fs]; VAF 1.1%**

The variant is a frameshift mutation resulting in a premature stop codon. This leads either to an early termination of protein synthesis or to nonsense-mediated mRNA decay, where the defective mRNA is prematurely degraded. *BAP1* is a tumor suppressor and deubiquitinating enzyme. *TET2* encodes for a tumor suppressor and DNA demethylase, is frequently mutated in hematologic malignancies.

***FGFR2-BICC1* Fusion**

The *FGFR2*-*BICC1* fusion has been found in cholangiocarcinoma ([PMID: 28034880](https://www.ncbi.nlm.nih.gov/pubmed/28034880)). Expression of this fusion in cell lines demonstrated activation of the MAPK pathway, increased cellular proliferation and anchorage-independent growth of cells (GOF, gain-of-function) ([PMID: 24122810, 23558953](https://www.ncbi.nlm.nih.gov/pubmed/24122810,23558953)). The pan-FGFR-targeted inhibitors pemigatinib and futibatinib are FDA-approved for the treatment of patients with *FGFR2*-fusion positive cholangiocarcinoma. In a Phase I trial, three patients with intrahepatic cholangiocarcinoma harboring an *FGFR2*-*BICC1* fusion were treated with futibatinib and demonstrated partial response with progression-free survivals of 15.9 months, 4.4 months and 12.7 months, respectively ([PMID: 34551969](https://www.ncbi.nlm.nih.gov/pubmed/34551969)).
